# Restoring Meropenem Activity Against VIM‐1‐Producing *Klebsiella* *pneumoniae* Through Zinc‐Binding Ligands

**DOI:** 10.1002/cbic.70509

**Published:** 2026-08-26

**Authors:** Chiara Ragusa, Ruqaiya Khalil, Valentina Giglio, Kaveh Eskandari, Fraser J. Scott, Attilio V. Vargiu, Graziella Vecchio

**Affiliations:** ^1^ Dipartimento di Scienze Chimiche Università degli Studi di Catania Catania Italy; ^2^ Department of Physics University of Cagliari Cagliari Italy; ^3^ Institute of Biomolecular Chemistry CNR‐ICB Catania Italy; ^4^ Department of Pure and Applied Chemistry University of Strathclyde Glasgow Scotland

**Keywords:** antibiotics, carbapenem, carbonic anhydrase, chelation, chemistry, docking (molecular), *K* *lebsiella pneumoniae*, meropenem, microbiology, molecular dynamics

## Abstract

The growing prevalence of metallo‐β‐lactamase (MBL)‐producing bacteria threatens the clinical efficacy of carbapenems such as meropenem. To investigate potential strategies for restoring β‐lactam activity, a panel of compounds was evaluated as putative MBL inhibitors and antibiotic adjuvants. The panel included FDA‐approved drugs (carbonic anhydrase inhibitors, an antiviral agent, and a metal chelator), and two novel histidine–pyridine derivatives (D‐ and L‐2‐amino‐3‐(1H‐imidazol‐4‐yl)‐N‐[2‐(pyridin‐2‐yl)ethyl]propenamides). The latter were synthesized and characterized by NMR spectroscopy and mass spectrometry. Combination assays against Verona Integron–encoded Metallo‐β‐Lactamase 1 (VIM‐1)–producing *Klebsiella pneumoniae* showed that D‐penicillamine and chlorpropamide restored meropenem activity. These effects were supported by time‐dependent inhibition and checkerboard assays. Molecular docking and MD simulations with the VIM‐1 enzyme further elucidated the putative binding modes of these inhibitors. These results suggest that repurposing the conventional D‐penicillamine and chlorpropamide drugs can effectively counteract MBL‐mediated resistance.

## Graphical abstract

Approved zinc‐binding drugs and newly synthesized HisPyr ligands were evaluated as adjuvants to meropenem against metallo‐β‐lactamase‐producing *Klebsiella pneumoniae*. Chlorpropamide and D‐penicillamine restored meropenem activity against the VIM‐1‐producing strain, while computational analyses supported interactions with the enzyme's binuclear zinc active site, highlighting drug repurposing as a potential strategy against carbapenem resistance.

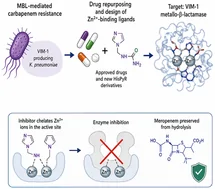

## 1 Introduction

The indiscriminate use of antibiotics in both agriculture and healthcare has substantially contributed to the rise of drug‐resistant pathogenic microorganisms [1, 2]. Among various antibiotic classes, particular attention is given to the development of resistance against β‐lactams [3, 4]. These drugs are commonly used to treat bacterial infections and account for over 50% of all injectable antibiotic prescriptions in the USA and Europe [5, 6] and are of critical importance in clinical settings. Their popularity stems from their broad‐spectrum activity which targets a wide range of bacterial species and the fact that they are generally well‐tolerated with minimal side effects [7]. However, their extensive use has accelerated the development of antibiotic resistance (AR), which is mostly mediated by the production of β‐lactamases, enzymes that hydrolyze the four‐membered ring of β‐lactams [7]. Four classes of β‐lactamases are recognized based on their catalytic mechanism and amino acid sequence: A, C, and D, collectively known as serine‐β‐lactamases (SBLs), and B, comprising metallo‐β‐lactamases (MBLs) [8, 9]. MBLs are a family of proteins with one or two Zn^2+^ ions in their active site [10]. The most important and commonly reported MBLs are: VIM (Verona Integron‐encoded Metallo‐β‐lactamase), NDM (New Delhi Metallo‐β‐lactamase), and IMP (Imipenemase). They cause high‐level resistance to carbapenem antibiotics and spread easily between bacteria, making infections difficult to treat. Current SBL inhibitors approved by the Food and Drug Administration (FDA) include clavulanic acid, tazobactam and sulbactam [11], which are routinely used in combination therapies to treat infections caused by SBL‐producing pathogens [12]. However, to date, no MBL inhibitors are available for clinical use [13]. This gap is particularly concerning given the rising incidence of carbapenem resistance as carbapenems are commonly the first‐line medications for infections caused by multidrug‐resistant bacteria [14, 15, 16]. In recent years, bicyclic boronates such as taniborbactam or xeruborbactam, have been studied as MBL inhibitors [17, 18]. The combination of antibiotic/MBL inhibitor such cefepime‐taniborbactam has complited phase 3 clinical trial (CERTAIN‐1; NCT03840148) [19] and demonstrates high *in vitro* activity against MBL isolates [20, 21].

**FIGURE 1:**
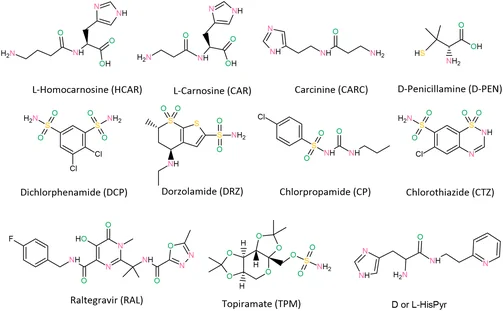
Chemical structures of natural and synthetic zinc‐binding compounds evaluated as potential MBL inhibitors. The panel includes natural zinc biochelators (L‐homocarnosine, L‐carnosine, D‐carcinine), clinically approved carbonic anhydrase (CA) inhibitors (dichlorphenamide, chlorphenamide, dorzolamide, chlorothiazide, and topiramate), and the clinically approved drugs (raltegravir and D‐penicillamine).

In light of the growing threat posed by MBL‐producing bacteria, repurposing existing drugs has emerged as a promising strategy for the development of new antimicrobial agents [22, 23]. This approach identifies novel therapeutic applications beyond the original medical use of pharmaceutical compounds [24]. Importantly, such drugs have already been extensively studied in humans, providing well‐established safety, efficacy, pharmacokinetic and toxicity profiles. Consequently, this pre‐existing data allows a deeper comprehension of the drugs’ pharmacology, optimal routes of administration, and the establishment of an appropriate dosing regimens [25, 26]. Another relevant advantage of repurposing is its potential to significantly reduce development costs and timelines, bypassing certain phases of traditional clinical trials [24, 27, 28]. In this spirit, a panel of compounds was examined in this work for their inhibitory activity against MBLs, including clinically approved carbonic anhydrase inhibitors (dichlorphenamide, dorzolamide, chlorothiazide, topiramate [29], and chlorpropamide); the antiviral drug raltegravir [30]; the copper chelator D‐penicillamine [31]; and the naturally occurring zinc biochelators carnosine, carcinine, and homocarnosine [32, 33, 34]. In addition, based on the efficacy showed by polypiridine and polyimidazole derivatives as MBL inhibitors [35, 36], we synthesized two new metal chelators, L‐ and D‐histidine derivatives with aminoethylpyridine (Figure 1).

The rationale for selecting these compounds is their ability to chelate Zn^2+^ within the active site of MBLs, thereby inhibiting enzyme activity and restoring antibiotic efficacy [37]. Histidine‐containing dipeptides (e.g., carnosine and its analogs) offer safe, selective, and biocompatible zinc binding [38], while carbonic anhydrase inhibitors are FDA‐approved drugs with optimized pharmacological profiles [39] and possess structural motifs that effectively coordinate zinc in the catalytic site of the carbonic anhydrase enzyme (often via sulfonamide or similar groups) [40, 41].

We tested the ability of these compounds to restore the antibacterial activity of the carbapenem antibiotic meropenem (MEM) against New Delhi Metallo β‐Lactamase 1 (NDM‐1) and Verona Integron–encoded Metallo‐β‐Lactamase 1 (VIM‐1) producing *K. pneumoniae*. Compounds showing inhibitory potential were further assessed through growth kinetics and checkerboard assays. Notably, D‐penicillamine (hereafter D‐Pen) and chlorpropamide (hereafter CP) fully restored meropenem efficacy against the VIM‐1‐producing stain but not the NDM‐1‐producing strain. Furthermore, we investigated the most likely binding modes of CP and L‐HisPyr against VIM‐1 by means of molecular docking and molecular dynamics (MD) simulations. Our results are consistent with stable binding modes involving Zn^2+^ coordination and interactions with catalytically relevant residues, supporting the restoration of meropenem activity observed *in vitro*.

## 2 Results and Discussion

D‐ and L‐2‐amino‐3‐(1H‐imidazol‐4‐yl)‐N‐[2‐(pyridine‐2‐yl)‐ethyl] propenamide (HisPyr) were synthesized as potential inhibitors of MBLs. Pyridine and imidazole are well‐known ligands for Zn^2+^, and polypyridine and polyimidazole ligands have been evaluated as MBL inhibitors [42, 43]. We synthesized L‐ and D‐HisPyr systems to investigate the activity of the different enantiomers.

D‐ and L‐ HisPyr were synthesized from 2‐(pyridine‐2‐yl) ethan‐1‐amine and D‐ and L‐histidine with protected amino group, as shown in Figure S1.

These compounds were characterized by NMR spectroscopy and ESI‐MS spectrometry (Figures S2–S8). The ^1^H NMR spectra of Boc‐ protected enantiomers confirmed the condensation between His and the pyridinylamine moiety. In the aromatic region, the H‐2 and H‐5 signals of the imidazole ring resonate at 6.75 and 7.51 ppm, and the pyridine protons resonate at 7.66, 7.19, and 7.14 ppm. These signals are observed alongside the ABX system of His and the diastereotopic ethylenic chain protons. The ^1^H NMR spectra of the deprotected derivatives showed changes in the chemical shifts of the aliphatic CH_2_CH protons (ABX spin system) of His compared with the protected molecule (Figure S7). The identity of the conjugates was confirmed by ESI HR MS (Table S1, Figure S8). The +ESI‐HRMS spectrum of L‐HisPyr, reported in Figure S8A, shows the peak of the molecular ion [M+H]^+^ at *m*/*z* 260.1506 and the single‐charged sodium adduct [M+Na]^+^ at *m*/*z* 282.1326. Additional dimeric species at higher molecular weight are also present, including the ion at *m*/*z* 541.2759, assigned to [2M+Na]^+^. A similar distribution of species was observed for D‐HisPyr, as expected (Figure S8B).

### 2.1 In Vitro Activity Against NDM‐1‐ and VIM‐1‐Producing *K. pneumoniae*

An initial screen of synergy was conducted to evaluate the ability of synthesized compounds and clinically approved drugs to restore meropenem (MEM) activity. The adjuvants were tested at 500 μM to maximize the likelihood of detecting synergistic interactions at the preliminary stage. Bacterial growth was measured with MEM at 4 μM, a concentration with no antibacterial effect, combined with potential adjuvants at 500 μM against *K. pneumoniae* strains producing either NDM‐1 (NCTC 13443) or VIM‐1 (NCTC 13440) MBLs. Such cellular systems are indeed informative, since restoration of MEM activity in intact bacteria reflects inhibition of metallo‐β‐lactamase activity under physiological conditions, while also accounting for factors such as periplasmic access, membrane permeability, and efflux that are not captured in purified‐enzyme assays.

**FIGURE 2:**
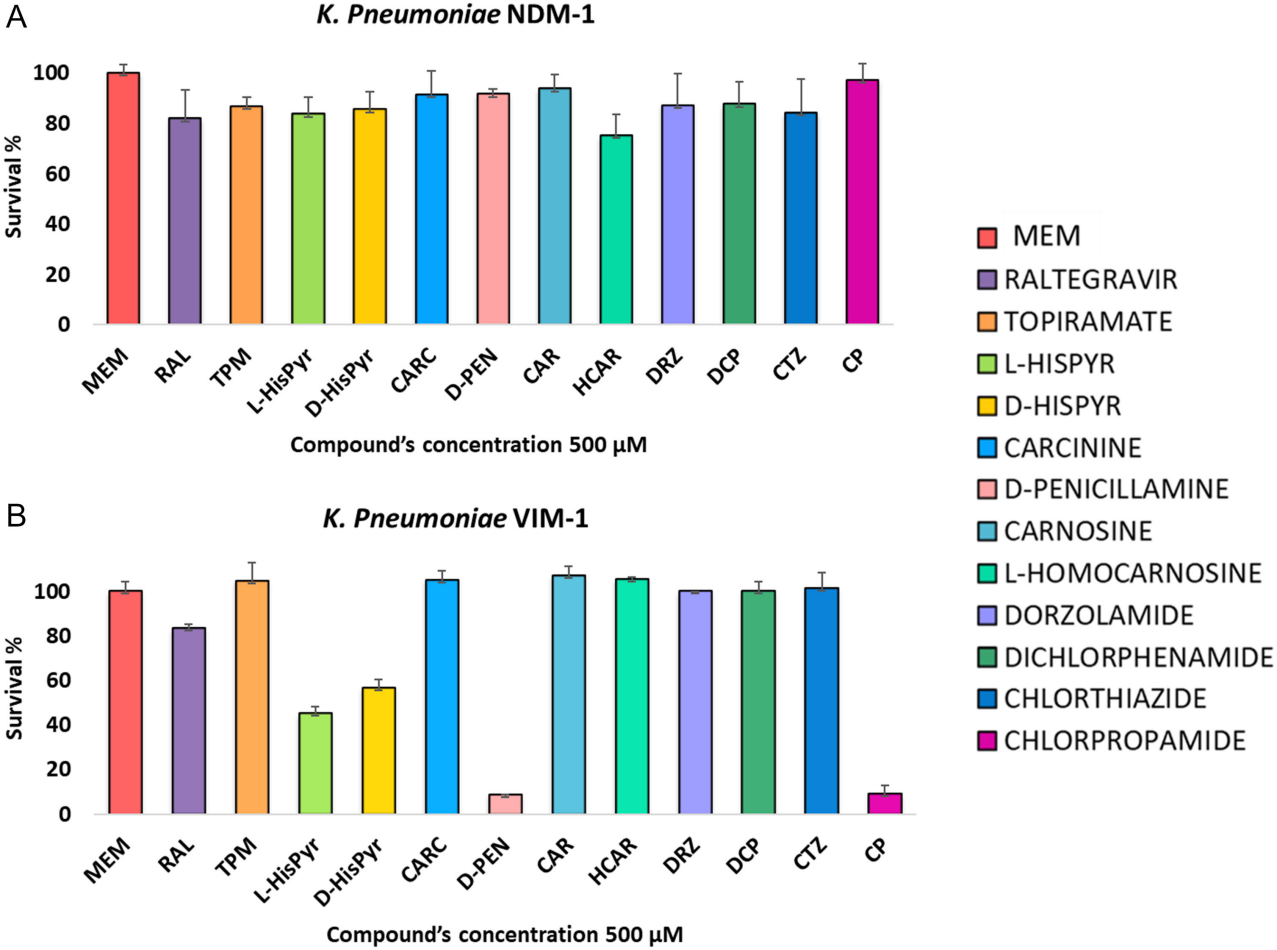
(A,B) Synergistic assays on NDM‐1 (A) and VIM‐1 (B) producing *K. pneumoniae* strains. In both assays, meropenem and the adjuvant were used at concentrations of 4 and 500 μM, respectively.

For the NDM‐1‐producing strain, none of the tested compounds (Figure 1) restored MEM activity (Figure 2A). In contrast, VIM‐1‐producing *K. pneumoniae* showed differential responses to the tested compounds (Figure 2B). D‐HisPyr and L‐HisPyr each restored approximately 50% of MEM antibacterial activity at 500 μM. Notably, D‐penicillamine (D‐Pen) and chlorpropamide (CP) restored MEM activity. This enzyme selectivity could possibly be explained by the greater conformational plasticity of VIM‐1 compared with NDM‐1, particularly in Loop 3 and Loop 10, which undergo substantial conformational changes upon ligand binding [44, 45]. This flexibility allows VIM‐1 to accommodate structurally diverse inhibitors while maintaining catalytic efficiency toward β‐lactam substrates [46]. The potential inhibition of VIM‐1 but not NDM‐1 suggests that inhibitor design strategies should consider enzyme‐specific structural features rather than relying on universal zinc chelation alone.

**FIGURE 3:**
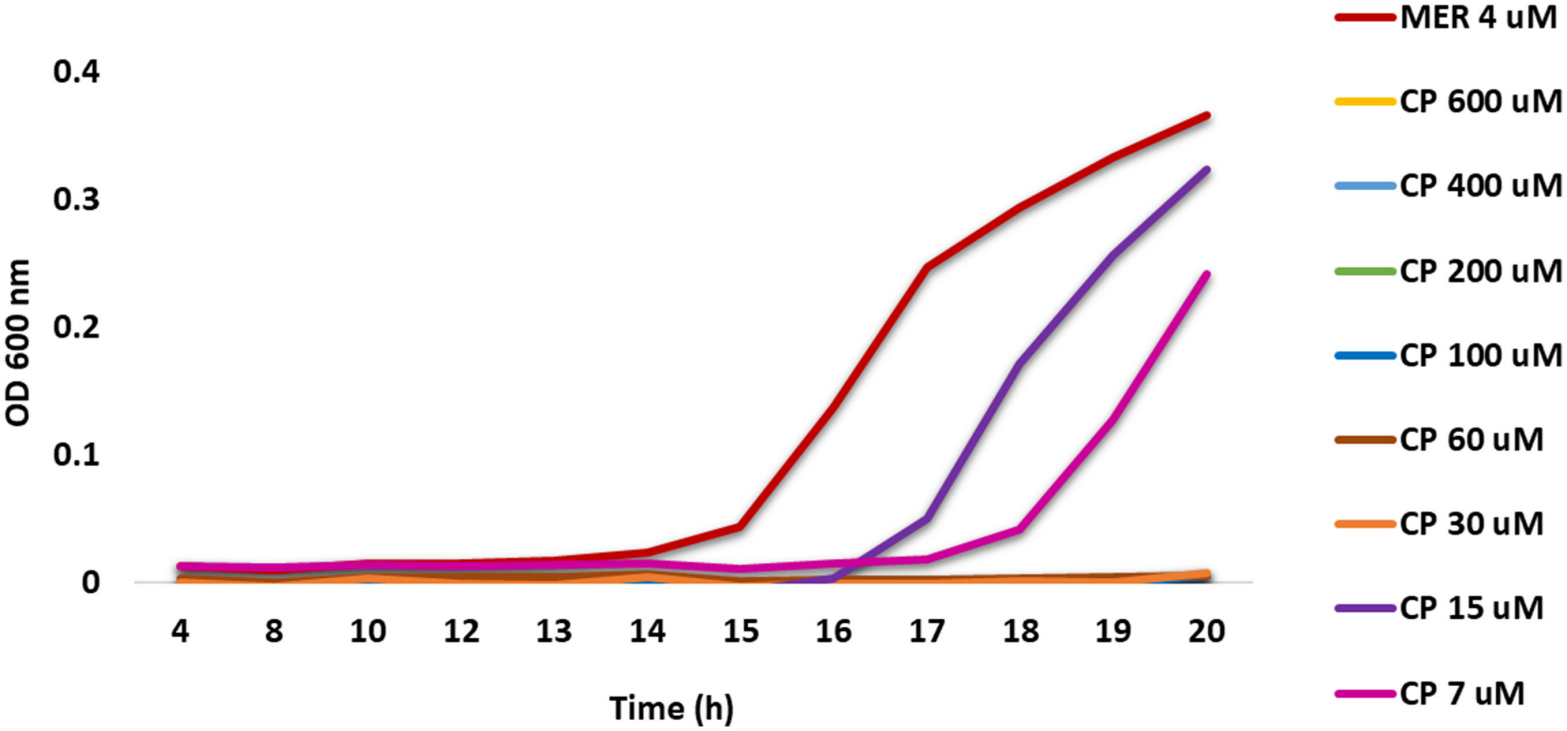
Time‐dependent inhibition test of *K. pneumoniae*‐producing VIM‐1 using different concentrations of CP co‐administered with MEM (4 μM).

**FIGURE 4:**
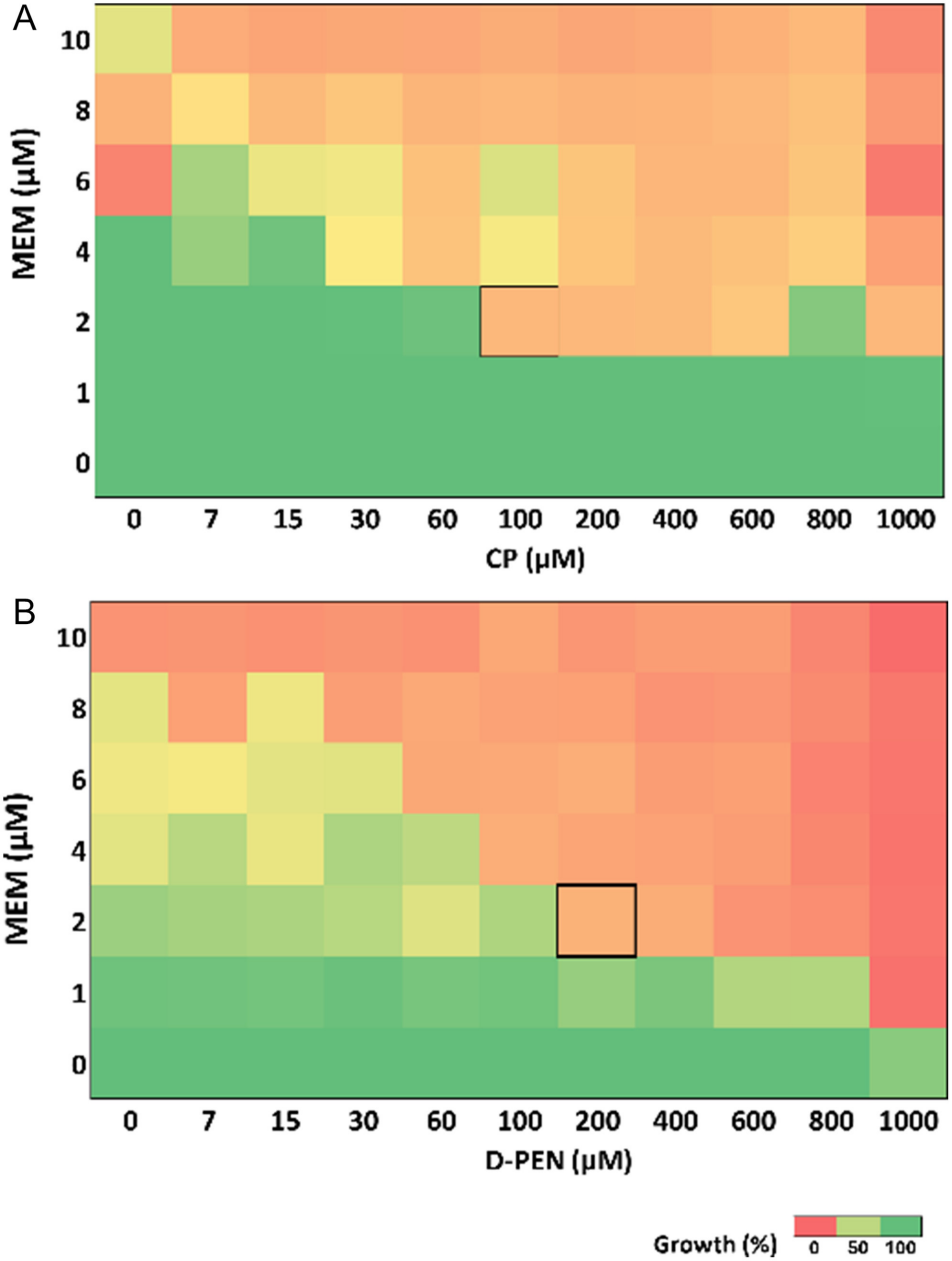
Checkerboard synergy assays of meropenem combined with chlorpropamide (CP) and D‐penicillamine (D‐Pen) against VIM‐1‐producing *K. pneumoniae*.

To investigate the synergistic interactions observed in the initial screening, time‐dependent inhibition assays were first performed. These experiments revealed a concentration‐dependent reduction in bacterial growth when CP or D‐Pen were combined with MEM (Figures 3 and S9). Synergistic interactions were then assessed using checkerboard assays, in which multiple concentrations of MEM were combined with varying concentrations of the inhibitors. The Fractional Inhibitory Concentration Index (FICI) was calculated as described in the Methods section with values <0.5 indicating synergistic activity. D‐Pen exhibited synergistic interactions with FICI values <0.40 (Figure S10), while CP displayed FICI values <0.43 (Figure 4). Because these drugs exert their activity, at least in part, through Zn^2+^ binding, their repurposing should be assessed, given the potential for off‐target inhibition of human metal‐dependent enzymes. Indeed, selectivity for bacterial metalloenzymes over host metalloenzymes remains a major challenge in the development of metal‐binding inhibitors [47, 48].

While CP and D‐Pen both have well‐established pharmacokinetic and clinical safety profiles [49, 50], the suitability of these compounds for repurposing indeed depends on whether the concentrations associated with synergy are clinically achievable. In the case of CP, synergistic activity with MEM was observed at 30 µM (see Figure 3). The pharmacokinetic studies report peak plasma concentrations of approximately 27 µg/mL (ca. 100 µM) following a standard 250 mg oral dose, suggesting that the concentrations associated with synergy *in vitro* are within the range of clinically achievable systemic exposures [51]. In contrast, for D‐Pen the reported peak plasma concentrations following standard oral dosing are approximately 1–2 mg/L (7–13 µM), below the concentrations associated with synergistic activity in the present study (200 µM; see Figure S9) [52]. Consequently, D‐Pen may be better regarded as a mechanistic proof‐of‐concept inhibitor or a starting point for further optimization rather than an immediately repurposable adjuvant.

### 2.2 In Silico Assessment of Molecular Interactions Between Putative Inhibitors and MBLs

We further investigated the molecular determinants of the interactions between VIM‐1 and the compounds that restored MEM activity against VIM‐1 producing K. pneumoniae strain. We first performed molecular docking calculations. Tables 1 and S2 report the average and pose‐wise data on the affinity and the key interactions for all compounds. Although D‐Pen showed strong in vitro activity, its small molecular size is likely to favor the occurrence of nonspecific Zn^2+^ chelation [53] rather than stable binding within the enzyme active site. In agreement with this hypothesis, docking results showed a weak and diffused interaction profile of this compound at the active site (Figure S11). For this reason, D‐Pen is not discussed further in the following sections. Furthermore, D‐HisPyr and L‐HisPyr displayed an almost identical docking profile (Figure S12). As both enantiomers share the same functional groups and coordination features, we hereafter discuss only L‐HisPyr as a representative compound.

**TABLE 1:** Docking results and key interactions for selected ligands (D‐Pen, CP, D‐HisPyr, and L‐HisPyr) targeting VIM‐1.

Ligand	Docking score, kcal/mol	Key interactions^a^
D‐Pen	−3.7 ± 0.2	H‐bonds: Asn210; Salt bridges: His179, His240; Chelates Zn^2+^
CP	−7.0 ± 0.1	H‐bonds: Asp118, Asn210; Hydrophobic: Trp87, Phe62
D‐HisPyr	−6.5 ± 0.5	H‐bonds: Asn210; Hydrophobic: Asp118, Phe62; Salt bridge: His179
L‐HisPyr	−6.3 ± 0.7	H‐bonds: Asn210, His201; Hydrophobic: Phe62, Asp118

a Key predicted interactions are derived from the top‐ranked docked pose.

**FIGURE 5:**
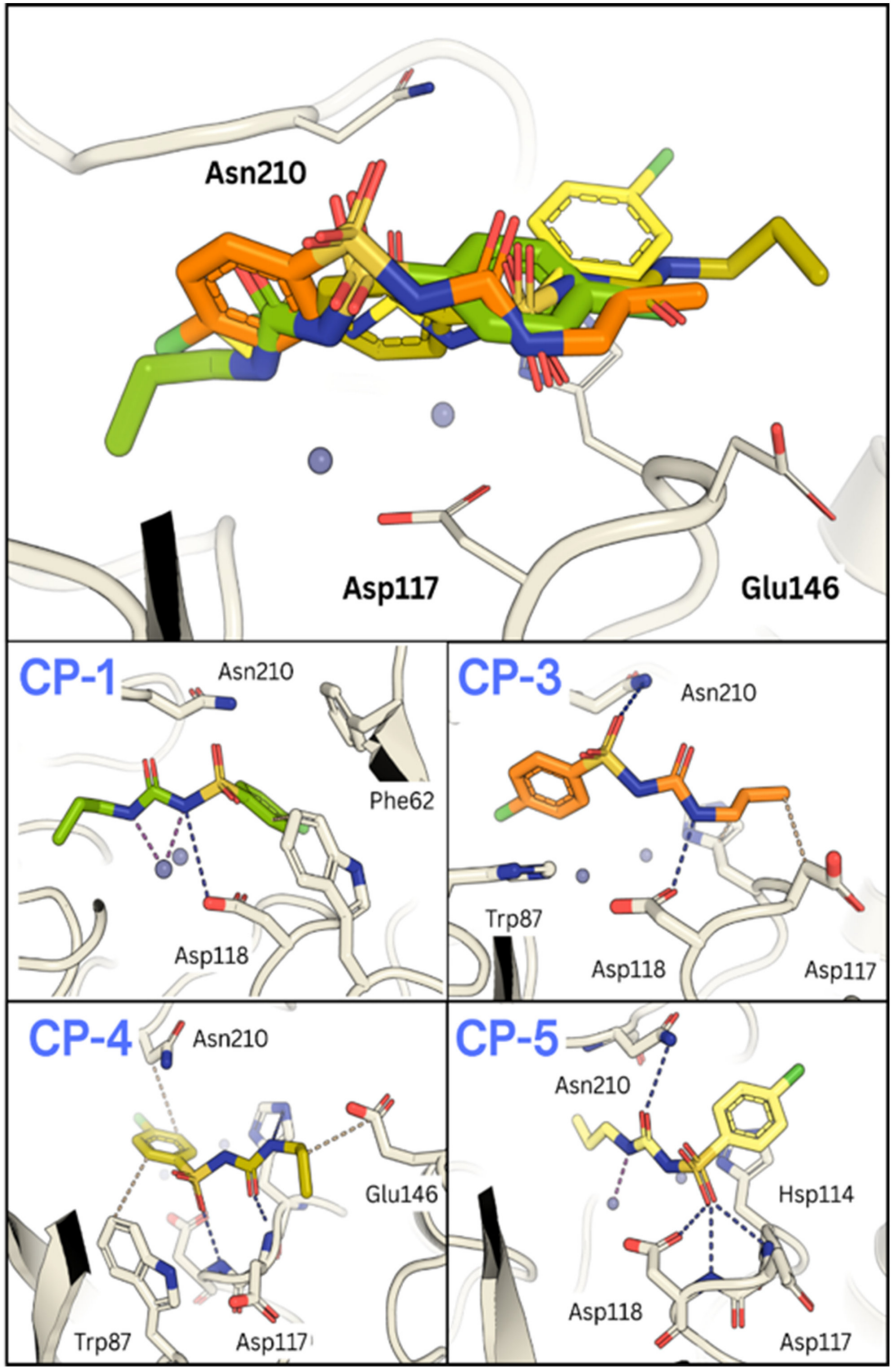
Overlay and comparison of the top‐ranked docking poses (poses 1–5) of CP within the active site of VIM‐1. The highest‐ranked pose (pose 1; green carbons, thickest sticks) substantially overlaps with pose 2 and thus represents both poses. Lower‐ranked poses (pose 3, orange; poses 4 and 5, yellow; progressively thinner sticks) exhibit minor variations in orientation‐while maintaining interactions with the crucial residues. Insets provide detailed interactions observed in individual poses, showcasing interactions with catalytically relevant residues: Asn210, Asp117, Asp118, Trp87, Phe62, Glu146, and His114, and zinc‐coordination (magenta dashes; Zn^2+^ depicted as spheres). Hydrogen bonds are indicated as blue dashed lines, and hydrophobic interactions as wheat‐colored dashed lines.

**FIGURE 6:**
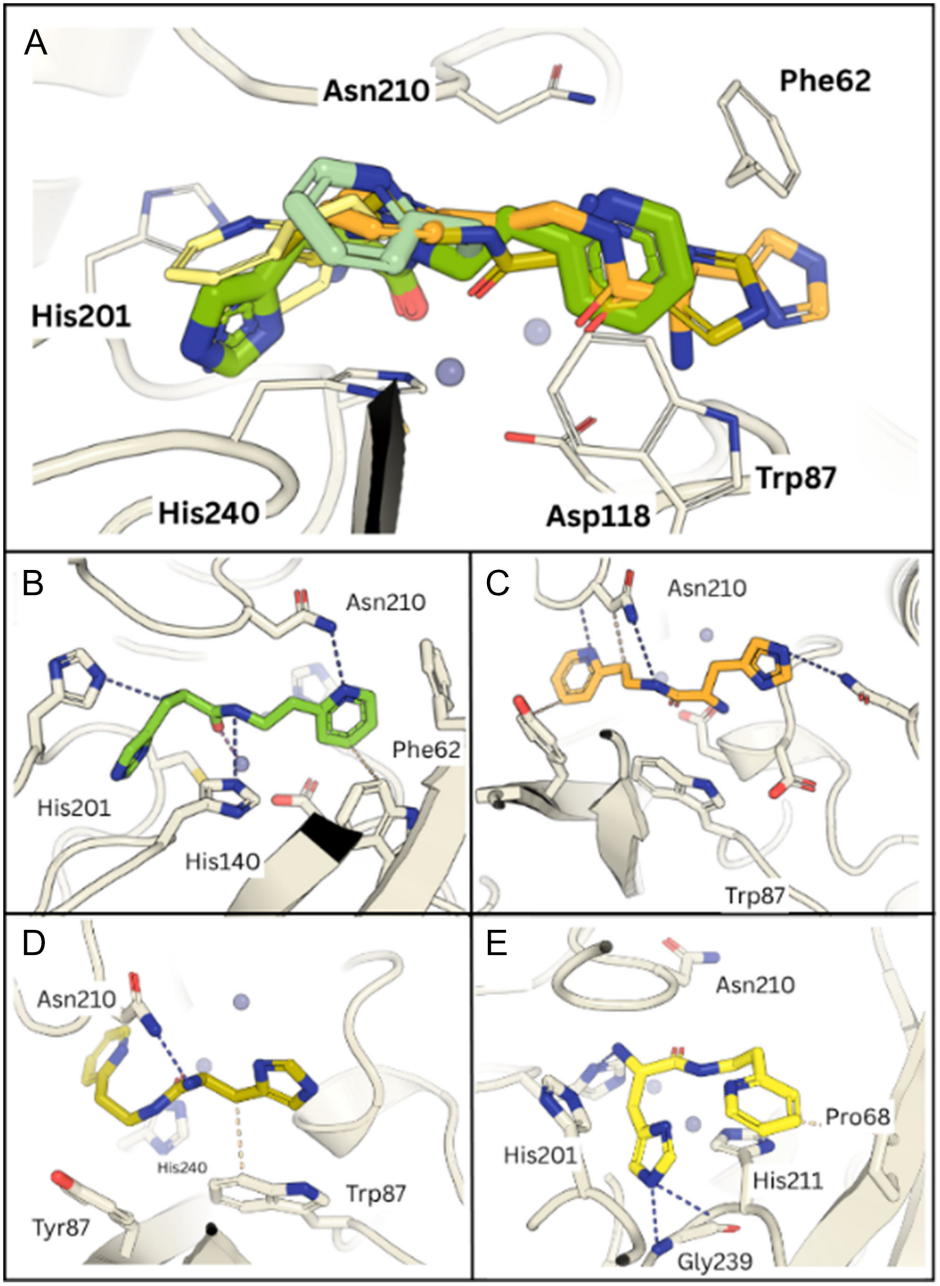
Overlay and comparison of the top‐ranked docking poses (poses 1–5) of L‐HisPyr within the active site of VIM‐1. The highest‐ranked pose (pose 1; green carbons, thickest sticks) substantially overlaps with pose 2 and thus represents both poses. Lower‐ranked poses (pose 3, orange; poses 4 and 5, yellow; progressively thinner sticks) exhibit minor variations in orientation‐while maintaining interactions with the crucial residues. Insets provide detailed interactions observed in individual poses, showcasing interactions with catalytically relevant residues: Asn210, Asp118, His201, His240, Trp87, Glu146, and Phe62, along with zinc coordination (Zn^2+^ shown as spheres). Hydrogen bonds are indicated as blue dashed lines, and hydrophobic interactions as wheat‐colored dashed lines.

The top 5 poses of CP and L‐HisPyr are shown in Figures 5 and 6. The docking calculations suggest that both compounds can be accommodated within the active site of VIM‐1 through multiple predicted interactions involving Asn210, Asp118, His240, and Trp87 (see Tables 1 and S3 for details of these interactions). In several high‐affinity poses obtained for both ligands, the compounds coordinate directly with catalytic Zn^2+^ ions, typically through interactions involving oxygen atoms. Importantly, different ligand arrangements are compatible with specific Zn^2+^ coordination involving a carbonyl oxygen of the ligands.

These results are in line with the heterogeneity of the active site of VIM enzymes, particularly VIM‐1, enabling accommodation of diverse substrates while maintaining broad catalytic efficiency [46]. To further address this structural plasticity, particularly within loops surrounding the active site, we performed a set of 5 independent MD simulations (each of 200 ns in length) of the VIM‐1‐CP and VIM‐1‐L‐HisPyr complexes starting from each of the top 5 docking poses obtained for each ligand.

We employed a protocol previously developed for MBL systems [54, 55, 56], which builds on the upgraded Amber force field (UAFF) with tailored parameters for residues coordinating with Zn^2+^.

The stability of the ligand–VIM–1 complexes and the status of the metal coordination were evaluated along the MD simulations using multiple structural metrics. Specifically, we evaluated the deviation of the ligand and the protein backbone and the fluctuation of the residues to assess global and local conformational stability (Figure S13). Additionally, we monitored the distance between Zn^2+^ and the coordinating residues at the H3 and DCH sites over time (Figure S14). Taken together, these metrics clearly demonstrate the stability of the protein and the complexes, both globally and at the binding site, and support the robustness of the simulation protocol. The retention of coordination geometry observed in the trajectories indicates that the force field can accurately replicate the behavior of Zn^2+^‐dependent active sites.

To further evaluate the presence of one or more preferred ligand binding modes under dynamic conditions, MD trajectories were subjected to a cluster analysis (performed using the ligand heavy atoms RMSD as a parameter, after alignment of the trajectories to the protein binding site; see Section 3 for details). Cumulative trajectories were used for clustering, ensuring a unified description of the conformational space sampled during the simulations.

**FIGURE 7:**
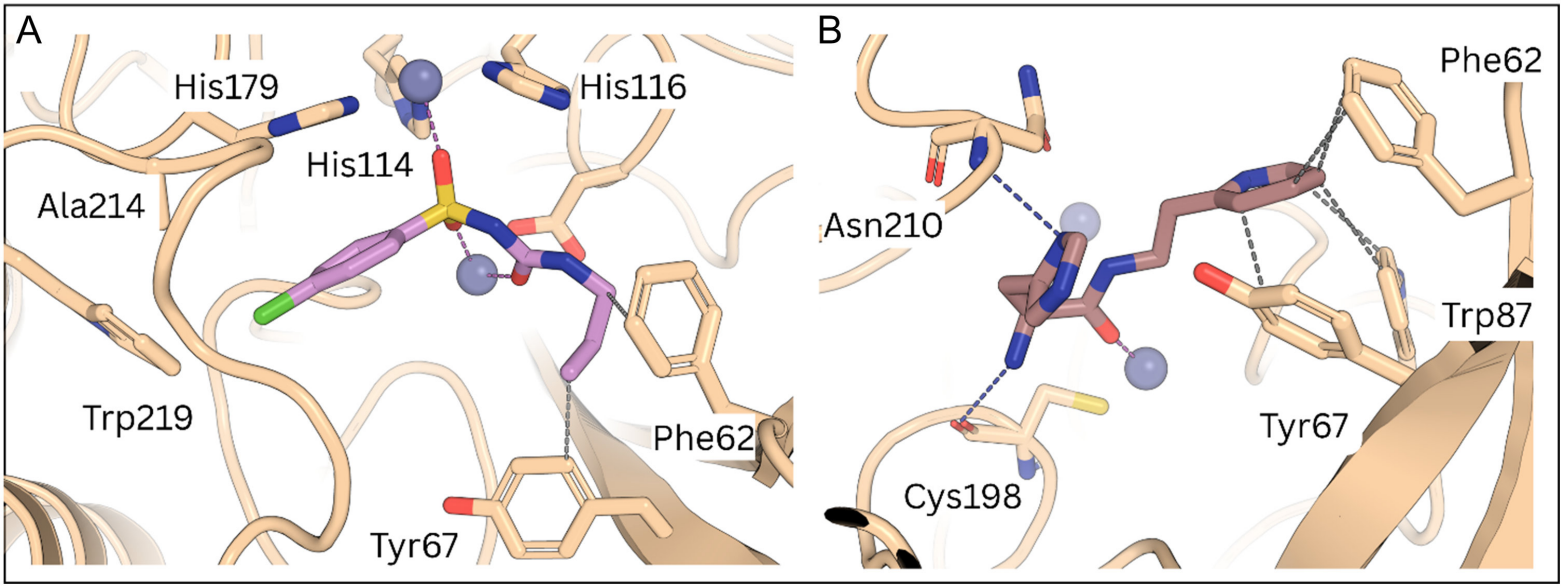
Most populated binding modes of CP (A) and L‐HisPyr (B) to the active site of VIM‐1, according to a cluster analysis performed on the cumulative trajectory obtained from five independent MD simulations of each complex. (A) CP binds near the binuclear zinc site, bonding with the residues of H3 site‐His114 and Asn210, Phe62, Thr252, and Val256. (B) L‐HisPyr binding at the Zn^2+^ site (DCH site), where it directly coordinates the zinc (magenta dashed line), and interacts with Cys198 and Asn210. Zinc ions are shown as gray spheres, ligands are colored magenta (CP) and brown (L‐HisPyr), and bonds are indicated with dashed lines (gray for hydrophobic, blue for hydrogen bond).

Analysis of the most populated clusters revealed stable and recurrent binding poses for both CP and HPI–HisPyr. Contact profiling of these dominant clusters was consistent with frequent interactions with Zn^2+^ ions and nearby residues, indicating a hybrid binding mode combining metal chelation and peripheral loop engagement. Importantly, the ligands exhibited contacts with catalytically relevant residues in all possible binding modes (Figure 7).

CP chelates the catalytic Zn^2+^ ion, using its sulfonyl and carbonyl oxygens to establish interactions with the Zn‐binding Histidines 114 and 116 (Figure 7A). CP also engages in hydrophobic interactions primarily with residues situated in Loop L3 (Phe62, Tyr67). Notably, Phe62 is a component of the hydrophobic patch surrounding the active site, where it contributes significantly to correct substrate positioning via hydrophobic interactions [44]. Residues of the hydrophobic patch, including Phe62, have been hypothesized to play crucial roles in substrate binding and enzyme stability [44, 57].

L‐HisPyr binds at the DCH site (Zn^2+^ site, see Figure S14) of VIM‐1, extending a metal coordination bond between its carbonyl oxygen (C = O) and the Zn^2+^ ion (Figure 7B). Additionally, L‐HisPyr forms hydrogen bonds with Asn210 and Cys198, contributing to a stabilized inhibitor–enzyme complex. Given the direct role of Asn210 in β‐lactam recognition, the interaction of L‐HisPyr with this residue implies potential competitive inhibition with natural substrates, leading to disruption of enzyme function [58]. This competitive inhibition hypothesis is supported by enzyme inhibition assays, where L‐HisPyr demonstrated inhibitory potential when tested in combination with meropenem, a carbapenem known to specifically interact with Asn210 [59].

The bi‐nuclear Zn center of VIM‐1 [and of all class B1 metallo‐beta‐lactamases] is essential for hydrolysis, and is a key target for inhibition [46, 60]. The interaction of the carbonyl oxygen in both CP and L‐HisPyr mimics the coordination of the β‐lactam carbonyl during catalysis, effectively blocking access to the catalytic metal center. By chelating Zn^2+^ through its carbonyl oxygen, the ligands disrupt catalysis, thereby halting β‐lactam hydrolysis.

## 3 Materials and Methods

### 3.1 Materials

1‐Hydroxybenzotriazole (HOBt), 1‐ethyl‐3 (3‐dimethylamino propyl) carbodiimide hydrochloride (EDC), trifluoroacetic acid, raltegravir, topiramate, carcinine, D‐penicillamine, dorzolamide, dichlorphenamide, chlorothiazide, chlorpropamide, and meropenem were purchased from TCI (Tokyo Chemical Industry co., Tokyo, Japan); Triethylamine (TEA), dimethyl sulfoxide (DMSO), carnosine, L‐homocarnosine, Nα‐Boc‐L‐histidine, Nα‐Boc‐D‐histidine, and 2‐(pyridine‐2‐yl) ethan‐1‐amine were purchased from Sigma‐Aldrich. Thin‐Layer chromatography (TLC) was carried out on silica gel plates (Merck 60‐F254).

Oxoid Agar Bacteriological and Oxoid Mueller‐Hinton Broth were purchased from Thermo Scientific.

### 3.2 NMR Spectroscopy

^1^H and ^13^C NMR spectra were recorded at 25 °C with a Varian UNITY PLUS‐500 spectrometer at 499.9 and 125.7 MHz, respectively. The NMR spectra were obtained using standard pulse programs from the Varian library. 2D experiments (COSY, gHSQCAD, gHMBC) were acquired using 1 k data points, 256 increments and a relaxation delay of 1.2 s.

### 3.3 ESI‐HR Mass Spectrometry

High‐resolution mass spectra were acquired using an Orbitrap Exploris Oe120 (Thermo Fisher) in positive electrospray ionization (ESI+) mode. Freshly prepared aqueous solutions of the conjugates were injected at a concentration of 10 μM. Sample solutions were injected into the ion source at a flow rate of 30 μL/min, with nitrogen as the desolvation gas. The electrospray capillary voltage was optimized to 2.5 kV to maintain adequate sensitivity and minimize in‐source fragmentation. The resolution obtained ranged from 45,000 to 70,000 FWHM. Mass spectra were processed with Xcalibur software (version 3.0). Each species is indicated by the *m*/*z* value of the most intense peak of its isotopic cluster.

### 3.4 Synthesis of D and L‐HisPyr

EDC (112 mg, 5.80 10^−4^ mol), HOBt (39 mg, 2.9 10^−4^ mol) and TEA (27 μL, 2.0 10^−4^ mol) were added to the Boc D‐ or L‐His (100 mg, 3.92 10^−4^ mol) solution in DMSO (1 mL). After 10 min, Pyr (47 μL, 3.9 10^‐ 4^ mol) was added to the mixture. The solution was stirred at room temperature for 24 h and the mixture was purified by flash chromatography using an RP C_18_ Aq. column and a linear gradient of water/methanol (0%–>100%). 40 mg of the final product were obtained (Yield 40%).

#### 3.4.1 Boc‐L‐HisPyr

^1^H NMR spectrum (500 MHz, D_2_O) *δ* (ppm): 8.31 (d, 1H, **2**), 7.66 (t, 1H, *J* = 7.7 Hz, **4**); 7.52 (s, 1H, **13**), 7.19 (d, 1H, *J* = 7.6 Hz, **3**), 7.15 (d, 1H, *J* = 7.8 Hz, **5**), 6.75 (s, 1H, **12**), 4.06 (dd, 1H, **9**), 3.43 (m, 1H, **6**, 3.43 (m, 1H, **7a**), 2.81 (m, 2H, **7b** and **10a**), 2.67 (dd, 1H, **10b**), 1.25 (s, 9 H, **16**).

^13^C NMR spectrum (125 MHz, D_2_O) *δ* (ppm): 181.5 (**14**), 173.8 (**8**), 158.1 (**1**), 157.0 (**11**,), 148.4 (**2**), 138.0 (**3**), 135.9 (**13**), 132.9 (**11**), 124.1 (**5**), 122.2 (**4**r), 81.4 (**15**), 54.9 (**10**), 38.8 (**6**), 36.3 (**7**), 28.9 (**9**), 27.5 (**16**).

#### 3.4.2 Boc‐D‐HisPyr

^1^H NMR spectrum (500 MHz, D_2_O) *δ* (ppm): 8.30 (d, 1H, **2**), 7.67 (t, 1H, *J* = 7.7 Hz, **4**); 7.53 (s, 1H, **13**), 7.21 (d, 1H, *J* = 7.5 **3**), 7.18 (d, 1H, *J* = 7.8 **5**), 6.74 (s, 1H, **12**), 4.07 (dd, 1H, **9**), 3.42 (m, 1H, **6**), 3.42 (m, 1H, **7a**), 2.83 (m, 2H, **7b** and **10a**), 2.68 (dd, 1H, **10b**), 1.24 (s, 9 H, **16**).

^13^C NMR spectrum (125 MHz, D_2_O) *δ* (ppm): 181.0 (**14**),174.0 (**8**), 158.5 (**1**), 157.3 (**11**),148.5 (**2**), 138.5 **3**), 136.2 (**13**), 133.9 (**12**), 124.4 (**4**), 122.5 (**5**), 81.5(**15**), 54.9 (**10**), 39.1 (**6**), 36.5 (**7**), 29.3 **9**), 27.7 (**16**).

The product Boc‐D‐HisPyr or B3oc‐L‐HisPyr has been treated with CF_3_COOH under stirring for 6 h to remove the Boc group. The final products were purified by Sephadex‐A25 DEAE (OH‐ form) to exchange trifluoroacetate ion.

#### 3.4.3 L‐HisPyr

^1^H NMR spectrum (500 MHz, D_2_O) *δ* (ppm): 8.23 (d, 1H, *J* = 4.9 Hz, **2**), 7.66 (t, 1H, *J* = 7.6 Hz, **3**); 7.52 (s, 1H, **13**), 7.13 (m, 1H, **4**), 7.15 (d, 1H, *J* = 7.0 Hz, **5**), 6.75 (s, 1H, **12**), 3.39 (m, 2H,**6**), 3.30 (m, 1H, 1H, **9**), 2.70 (t, 2H, *J* = 6.9 Hz, **7**), 2.67 (dd, 1H, *J* = 14.6, 7.2 Hz, **10a**) 2.59 (dd, 1H, *J* = 14.6, 6.8,**10b**).

ESI‐HRMS: *m*/*z* = [M+H]^+^ calcd for C_13_H_18_N_5_O 260.1506, found 260.1506; *m*/*z* = [M+Na]^+^ calcd for C_13_H_17_N_5_ONa 282.1325, found 282.1326; *m*/*z* = [2M+Na]^+^ calcd for (C_13_H_17_N_5_O)_2_Na 541.2758, found 541.2759.

#### 3.4.4 D‐HisPy

^1^H NMR spectrum (500 MHz, D_2_O) *δ* (ppm): 8.25 (d, 1H, *J* = 4.9 Hz, **2**), 7.60 (t, 1H, *J* 7.6 Hz, **3**); 7.52 (s, 1H, **13**), 7.14 (m, 1H, **4**), 7.03 (d, 1H, *J* = 7.86 Hz, **5**), 6.65 (s, 1H, **12**), 3.40 (m, 2H, **6**), 3.31 (dt, 1H, 1H, **9**), 2.71 (t, 2H, **7**), 2.66 (dd, 1H, *J* 14.6, 7.2 Hz, **10a**) 2.60 (dd, 1H, *J* = 14.6, 6.8, **10b**).

ESI‐HRMS: *m*/*z* = [M+H]^+^ calcd for C_13_H_18_N_5_O 260.1506, found 260.1505; m/z = [M+K]^+^ calcd for C_13_H_17_N_5_OK 298.1065, found 298.1064; *m*/*z* = [2M+Na]^+^ calcd for (C_13_H_17_N_5_O)_2_Na, 541.2758, found 541.2757, [2M+ K]^2+^ calcd for (C_13_H_17_N_5_O)_2_K 557.2497, found 557.2496.

### 3.5 Bacterial Strains and Culture Conditions

The bacterial strains used in the present study were *K. pneumoniae* producing NDM‐1 (NCTC 13443) and *K. pneumoniae* producing VIM‐1 (NCTC 13440). These strains are resistant to meropenem.

All materials were previously autoclaved at 121 °C for 15 min. Bacteria were maintained at −80 °C in 30% glycerol. The strains were precultured in cation‐adjusted Mueller–Hinton Broth for 24 h at 37 °C on a shaker.

### 3.6 Antimicrobial Activity Assays

A broth microdilution method, using a sterilized 96‐well plate, was used to carry out the screening of MEM combined with adjuvants using a bacterial inoculum of 5 × 10^5^ CFU/mL, according to the Clinical and Laboratory Standards Institute (CLSI, 30th edition) guidelines. For the fixed‐concentration screen, the compounds were screened at a concentration of 500 μM in combination with MEM at a concentration of 4 μM. The optical density measurements were determined using a UV spectrophotometer, Tecan Infinite M Nano+ microplate reader at 600 nm. For the time‐dependent studies, the experimental protocol was identical, except that CP and D‐pen were evaluated in a concentration range of 7–600 μM with MEM 4 µM, and optical density readings were taken every 60 s.

### 3.7 Checkerboard Assay

Dilution series of both MEM and D‐Pen were prepared in CAMHB from stock solutions (10^−2^ M) in a sterilized 96‐microplate. To evaluate synergistic activity, increasing concentrations of MEM were distributed across columns 2–12, while increasing concentrations of D‐Pen were distributed down the rows (rows B–H). Then, the bacterial suspension (ca. 10^5^ CFU/mL) was added to each well. Each well had a final volume of 200 µL, consisting of 100 µL of bacterial suspension and 100 µL of CAMHB containing the appropriate concentrations of MEM and D‐Pen. After overnight incubation at 37 °C, the optical density of the bacterial suspensions was measured at 600 nm using a Tecan plate reader. The values obtained were transformed into growth inhibition compared to untreated control wells, and the fractional inhibitory concentration index (FICI) was calculated using the following formula:

$$FICI\;=\frac{\text{MIC}\;of\;MEM\;in\;combination}{\text{MIC}\;of\;MEM\;alone}+\frac{MIC\;of\;compound\;in\;combination}{MIC\;of\;compound\;alone}$$

FICI values ≤0.5 were interpreted as synergistic interactions, values between 0.5 and 1 as additive effects, values between 1 and 4 as indicating indifference, and values >4 as antagonistic effects. When the MIC exceeded the highest concentration tested in the dilution series, the maximum tested concentration was used for the FICI calculation, and the result was reported as less than or equal to the obtained value.

### 3.8 *In Silico* Studies

Here, we describe the method and workflow for the computational experiments performed in this study. Initially, molecular docking simulations were carried out using AutoDock Vina 1.2.5 [61] to establish the binding mode of selected compounds toward the metallo‐β‐lactamase VIM‐1. Next, we performed MD simulations of the complexes formed by this enzyme with the two representative compounds CP and L‐HisPyr. Considering the inherent flexibility of the MBL binding site, particularly the motion of Loop 3 [demonstrated in both MD simulation [62] and NMR studies [63]] and Loop 10 upon ligand binding [64] and the limitations of rigid docking predictions [65], we initiated MD simulations from 5 distinct docking poses for both compounds. Each of these docking‐derived starting conformations is hereafter referred to as a 'seed’ (e.g., CP1 corresponds to the top‐ranked docking pose of CP, CP2 to the second‐ranked docking pose, and similarly HP1, HP2, etc., for L‐HisPyr).

Each seed was subjected to 200 ns of production MD simulations (totaling 1 μs across the five trajectories). Next, a cluster analysis was performed on the cumulative trajectory of each protein–ligand complex using the *cluster* command in CPPTRAJ. The analysis was based on mass‐weighted RMSD calculations of heavy atoms only, using the distance matrix metric (*dme*). This approach allowed the identification of dominant conformational states by grouping similar structures sampled during the simulation into representative clusters. Moreover, the protein–ligand interaction pattern was also monitored as a function of time. This approach provided quantitative insights into the temporal and spatial stability of critical interactions during MD simulation. Further details are provided below.

### 3.9 Molecular Docking

Molecular docking was performed using Vina 1.2.5 and the AD4 scoring function [66] enhanced by the AutoDock4Zn (AD4Zn) force field [67]. AD4Zn extends the standard AD4 force field by incorporating specialized parameters for zinc coordination, including the TZ pseudoatom, which improves ligand docking accuracy for zinc metalloproteins.

The crystal structure of VIM‐1 was obtained from the Protein Data Bank (PDB, https://www.rcsb.org) [68] with PDB code 8PGP [69]. The protein structures were prepared using the pdb4amber module implemented in AmberTools24 [70]. The most populous alternate conformations were retained, and missing atoms were added. The protein was protonated using *reduce* [71].

The receptor PDBQT file was prepared using the *mk_prepare_receptor.py* script, followed by *zinc_pseudo.py*, which added two TZ‐Zinc pseudo‐atoms to the enzyme binding site. The positions of the TZ atoms and the corresponding Zn geometries are illustrated in Figure S15.

The chemical structures of the ligands were obtained from PubChem in SMILES format and imported into the Maestro interface [Schrödinger Release 2025‐1: Maestro, Schrödinger, LLC, New York, NY, 2025]. The compounds were protonated and energy minimized using the LigPrep module in Maestro. The structures were then converted into PDBQT format using the *prepare_ligand.py* script.

Affinity maps for docking were generated individually for each ligand using the *prepare_gpf4zn.py* script in conjunction with the modified force field definition file‐ AD4Zn.dat. The grid box was centered around the active site of VIM‐1(His114, His116, His179, His240, Asp118, and Cys198) (see Figure S15) to encompass the entire binding pocket, ensuring adequate space for ligand conformational flexibility and metal coordination and thus biologically relevant ligand sampling. A grid step of 0.375 Å was used, and a grid size of 40 × 40 × 40 points was employed for VIM‐1. The exhaustiveness parameter was set to 64 (default: 8), while other parameters were set to their default values. Results were analyzed visually using AutoDockTools. Protein–ligand interactions were characterized using the PLIP web server (version 2.3.0), which employs a rule‐based algorithm to identify interacting atoms and functional groups and evaluates them against set geometric criteria. Using the default distance and angular thresholds, PLIP was employed to identify hydrogen bonds, hydrophobic contacts, π‐stacking, and π–cation interactions at the atomic level [72].

### 3.10 MD Simulations

MD simulations were performed using GROMACS (2023.04) [73]. Zinc was treated with a nonbonded upgraded Amber force field (UAFF)‐ff14 SB [60] force field with redistributed partial charges and adjusted nonbonded terms on the zinc‐coordinating residues [55, 56], following the protocol validated for VIM‐2 by Güven et al. [74], implemented here in GROMACS rather than Amber.

### 3.11 System Preparation

MD simulations were carried out using the protein structure prepared previously for docking simulations. For MD simulations, the UAFF force field was used in GROMACS. Following Macchiagodena et al. [55, 56], the zinc‐coordinating residues were renamed according to their UAFF formats.

The top‐ranked poses of both CP and L‐HisPyr, after docking using Vina, were converted into pdb format using OpenBabel [75]. The hydrogens were added again using the Maestro interface in Schrödinger. Ligands were then parameterized using Gaussian16 [Gaussian, Inc., Wallingford CT (2016) GaussView 5.0. Wallingford, E.U.A.] and converted to GROMACS‐compatible format via ACPYPE [76]. Zinc coordination was not restrained by bonds but maintained through accurate electrostatic and Lennard–Jones parameters defined in UAFF. The protein–ligand complexes were solvated using the TIP3P water model in a cubic box. Each system was neutralized by adding Na^+^ counter ions with the *genion* tool in GROMACS. Ion parameters were derived from the ions.itp file provided by the AMBER14 SB force field. 11 Na^+^ ions were added to achieve electrical neutrality, and 11.628–11.637 water molecules were added to solvate the systems, depending on the exact box dimensions.

### 3.12 Simulation Parameters

For each system, a structural relaxation was first performed using the steepest descent method for 5000 steps, followed by heating of the system to 300 K in the NVT ensemble and equilibration using NPT ensemble. The simulations were carried out using the leap‐frog integrator with a time step of 2 fs. Long‐range electrostatic interactions were calculated using the Particle Mesh Ewald (PME) method with a real‐space cutoff of 0.8 nm. Temperature coupling was maintained at 300 K using the V‐rescale thermostat (*τ* = 0.2 ps), applied separately to the protein–ligand complex and the solvent‐ion group. Production MD simulations were conducted for 200 ns per seed, totaling 1 µs across 5 independent replicas.

### 3.13 Cluster Analysis

For clustering, cumulative trajectories were generated for each system, and all frames were aligned to the Cα atoms of the active‐site residues (Figure S2). Clustering was performed on the heavy atoms of the ligand using an agglomerative algorithm ligand [CPPTRAJ [77], epsilon = 3.0 Å, complete linkage, nofit].

## 4 Conclusions and Perspectives

This study identifies two clinically approved drugs, D‐penicillamine and chlorpropamide, as adjuvants capable of fully restoring meropenem activity against VIM‐1‐producing *K. pneumoniae*. Additionally, two newly synthesized HisPyr ligands demonstrate partial synergistic activity and represent promising scaffolds for further optimization.

*In silico* analyses support Zn^2+^ interaction as a central mechanism of inhibition and provide molecular insights into ligand–enzyme interactions. However, a key limitation of this approach is its specificity for MBL‐mediated resistance. Because the proposed mechanism relies on chelation or interaction with the active‐site Zn^2+^, it would not be expected to counteract resistance mediated by serine carbapenemases such as KPC‐2 or OXA‐48, which *K. pneumoniae* may also produce. Its utility is therefore likely restricted to infections in which an MBL is the principal resistance determinant.

Chlorpropamide (an oral antidiabetic used to treat type 2 diabetes mellitus) and D‐penicillamine (a chelator used for Wilson's disease) effectively restored the antimicrobial activity of meropenem in VIM‐1 producers. Although the active concentrations identified in this study remain in the low tens of micromolar range and therefore do not represent highly potent inhibitors, they demonstrate that clinically approved zinc‐binding drugs can restore meropenem activity against VIM‐1‐producing *K. pneumoniae*. In particular, chlorpropamide exhibited activity at concentrations approaching reported therapeutic plasma exposures, supporting its potential as a starting point for drug repurposing or medicinal chemistry optimization. These results highlight the therapeutic potential of zinc‐binding conventional drugs and underscore drug repurposing as a rapid and cost‐effective strategy to address antimicrobial‐resistant infections caused by metallo‐β‐lactamase producers.

## Funding

This work was supported by the Fondazione di Sardegna (CUP: F23C25000160007), the National Recovery and Resilience Plan (CUP: 85B17000360007 and CUP: J85B17000360007), and the National Institutes of Health (R01AI136799).

## Conflicts of Interest

The authors declare no conflicts of interest.

## Supporting information

Supplementary Material

## Data Availability

The data that supports the findings of this study are available in the supporting information of this article.
