# Supplementary material for: Restoring Meropenem Activity Against VIM‐1‐Producing Klebsiella pneumoniae Through Zinc‐Binding Ligands
Source: Chembiochem. 2026 Aug 26;27(16):e70509. doi: 10.1002/cbic.70509 (PMC13509051; doi:10.1002/cbic.70509)
Supplement: Supplementary file 1 [file CBIC-27-e70509-s001.pdf]

Restoring Meropenem Activity against VIM-1-Producing Klebsiella pneumoniae through Zinc-Binding Ligands

Chiara Ragusa,[a,#] Ruqaiya Khalil,[b,#] Valentina Giglio,[c] Kaveh Eskandari,[d] Fraser J. Scott,[d] Attilio V. Vargiu,[b,*] Graziella Vecchio[a,*]

[a] Dipartimento di Scienze Chimiche, Università degli Studi di Catania, Viale A. Doria 6, 95125 Catania, Italy

[b] Department of Physics, University of Cagliari, Cittadella Universitaria, SP n°8, 09042 Monserrato, CA, Italy

[c] Institute of Biomolecular Chemistry CNR-ICB, Via Paolo Gaifami 18, Catania, Italy

[d] Department of Pure and Applied Chemistry, University of Strathclyde, Glasgow G1 1XL, Scotland

# these authors contributed equally to this work

* address correspondence to vargiu@dsf.unica.it or gr.vecchio@unict.it

CHEMICAL SYNTHESIS & CHARACTERIZATION

Figure S1. Scheme of synthesis of L-HisPyr and its enantiomer D-HisPyr

Figure S2. ^{1}H NMR spectrum of L-BocHisPyr (D_2O, 500 MHz)

Figure S3. ^{1}H NMR COSY spectrum of L-BocHisPyr (D_2O, 500 MHz)

Figure S4. APT spectrum of L-BocHysPyr (D_2O, 125 MHz)

Figure S5. HSQC spectra of L-BocHysPyr (D_2O, 500 MHz)

Figure S6. HMQC spectra of L-BocHisPyr (D_2O, 500 MHz)

Figure S7. ^{1}H NMR spectrum of D-HisPyr (orange) and L-HisPyr (blue) (D_2O, 500 MHz)

Figure S8. HR ESI spectra of L-HisPyr (A) and D-HisPyr (B)

BIOLOGICAL ACTIVITY & SYNERGY ASSAYS

Figure S9. Time-dependent inhibition of K. Pneumonia-producing VIM-1 using different concentrations of D-Pen co-administered with MEM (4 µM)

Figure S10. Checkerboard assay of MEM with D-PEN

DOCKING RESULTS

Figure S11. Docking poses of D-pen in the active site of VIM-1. (A) Overlay of the top five docking poses, showing a highly similar binding orientation across all poses. (B) Close-up view of the top-ranked pose, highlighting coordination of the catalytic Zn^{2+} ion (grey sphere) primarily through the thiol and carboxylate groups of D-Pen.

Figure S12. Overlay of the docked binding poses of the two enantiomers of HisPyr within the active site of VIM-1. The L-enantiomer is shown in yellow, while the D-enantiomer is shown in magenta. Both ligands adopt comparable orientations within the catalytic pocket and interact with the Zn^{2+} center and surrounding residues, highlighting their similar binding modes despite opposite chirality. Key active-site residues and metal ions are shown in stick representation, with Zn^{2+} depicted as a sphere.

MOLECULAR DYNAMICS (MD) ANALYSIS

Figure S13. Structural stability analysis of the MD simulations of the VIM-1–CP and VIM-1–L-HisPyr complexes across five independent simulations (CP-1 to CP-5 and L-HisPyr-1 to L-HisPyr-5). (A–B) Root mean square fluctuation (RMSF) of protein residues; (C–D) RMSD of the protein Cα atoms; (E–F) RMSD of the ligand heavy atoms as a function of time.

Figure S14. Close-up views of the Zn^{2+} ions (Zn301 at the H3 site and Zn302 at the DCH site) along with key coordinating residues (A, B). The distances (nm) between Zn^{2+} ions and the coordinating residues from the H3 (C, E) and DCH (D, F) clusters across five independent MD simulations.

DOCKING SYSTEMS SETUP

Figure S15. Active site of VIM-1 highlighting key residues involved in zinc coordination. The Zn^{2+} ions are coordinated by His114, His116, His179, His240, Asp118, and Cys198. TZ pseudoatoms (white spheres) were introduced using the zinc_pseudo.py script to improve the representation of metal coordination and enhance docking accuracy in AutoDock4Zn.

SUPPLEMENTARY TABLES

Table S1. Species found and calculated for the L- and D-HisPyr conjugates in ESI-HRMS.

Table S2: Docking scores and ligand efficiency of selected ligands. The docking scores represent the binding affinity between each ligand and VIM-1, with lower values indicating stronger binding. The top five ranked poses for each ligand are reported.

Table S3: PLIP-identified interactions between Chlorpropamide (CP) and VIM-1 across docking poses CP1-CP5.

Table S4: PLIP-identified interactions between L-HisPyr and VIM-1 across docking poses lHP-1–lHP-5.

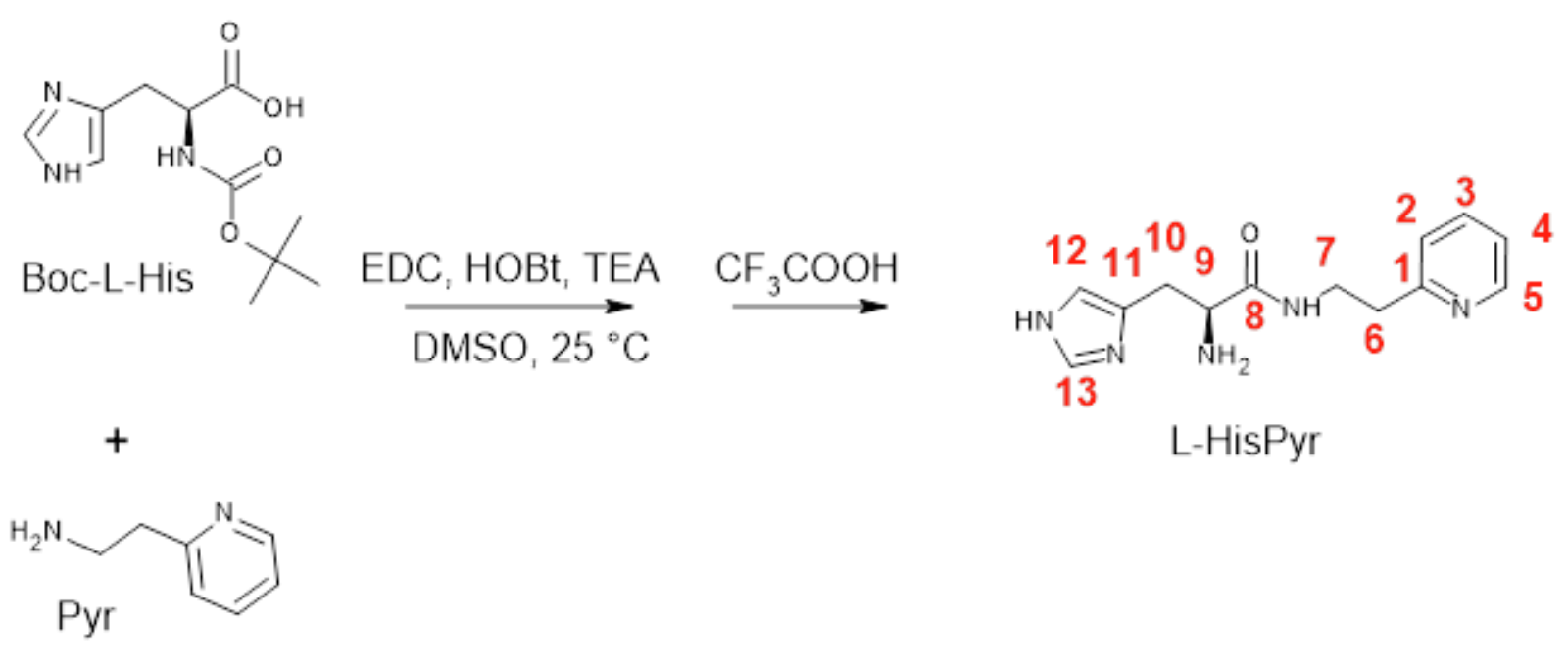

Figure S1. Scheme of synthesis of L-HisPyr and its enantiomer D- HisPyr

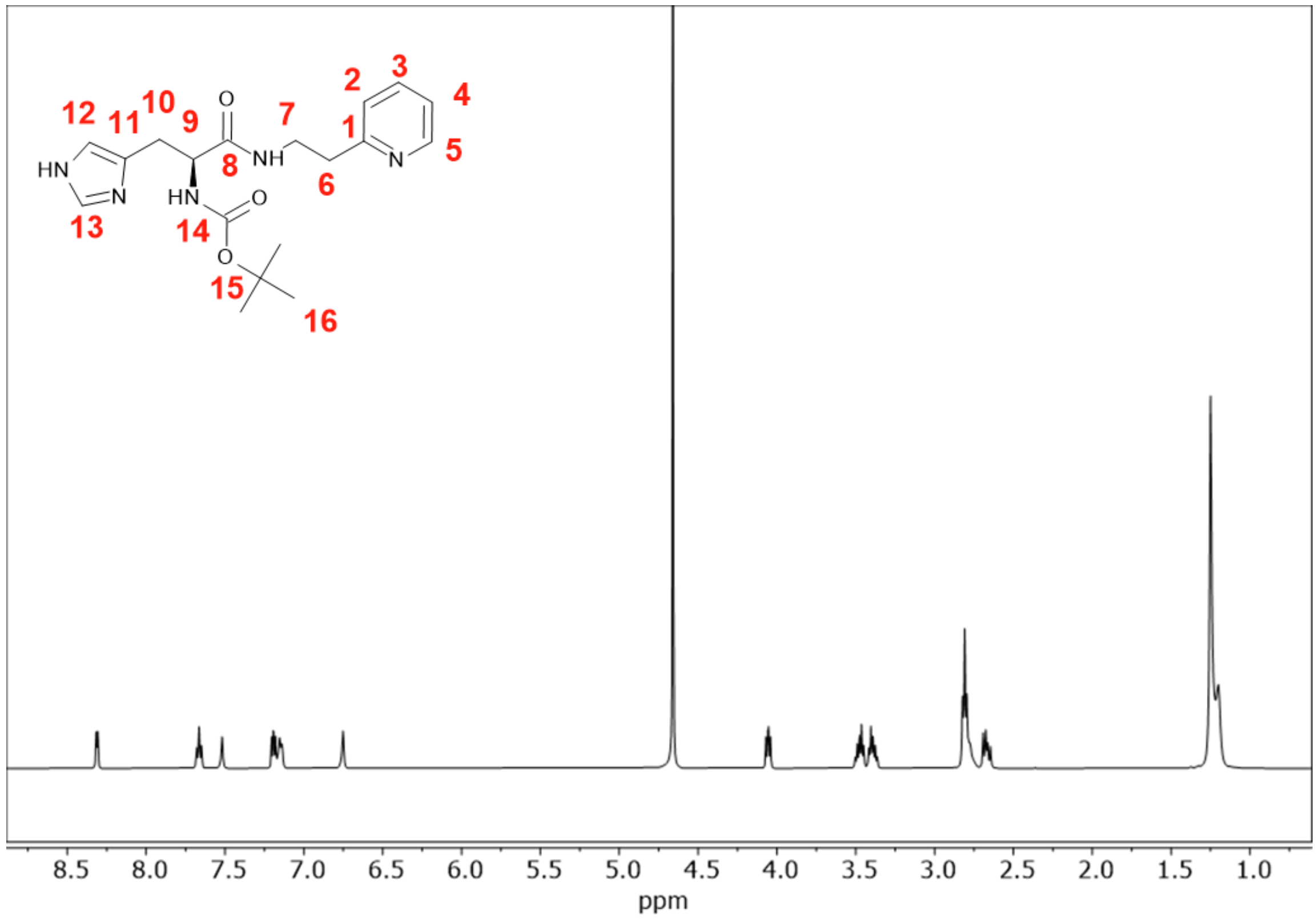

Figure S2.^{1}H NMR spectrum of L-BocHisPyr (D_2O, 500 MHz)

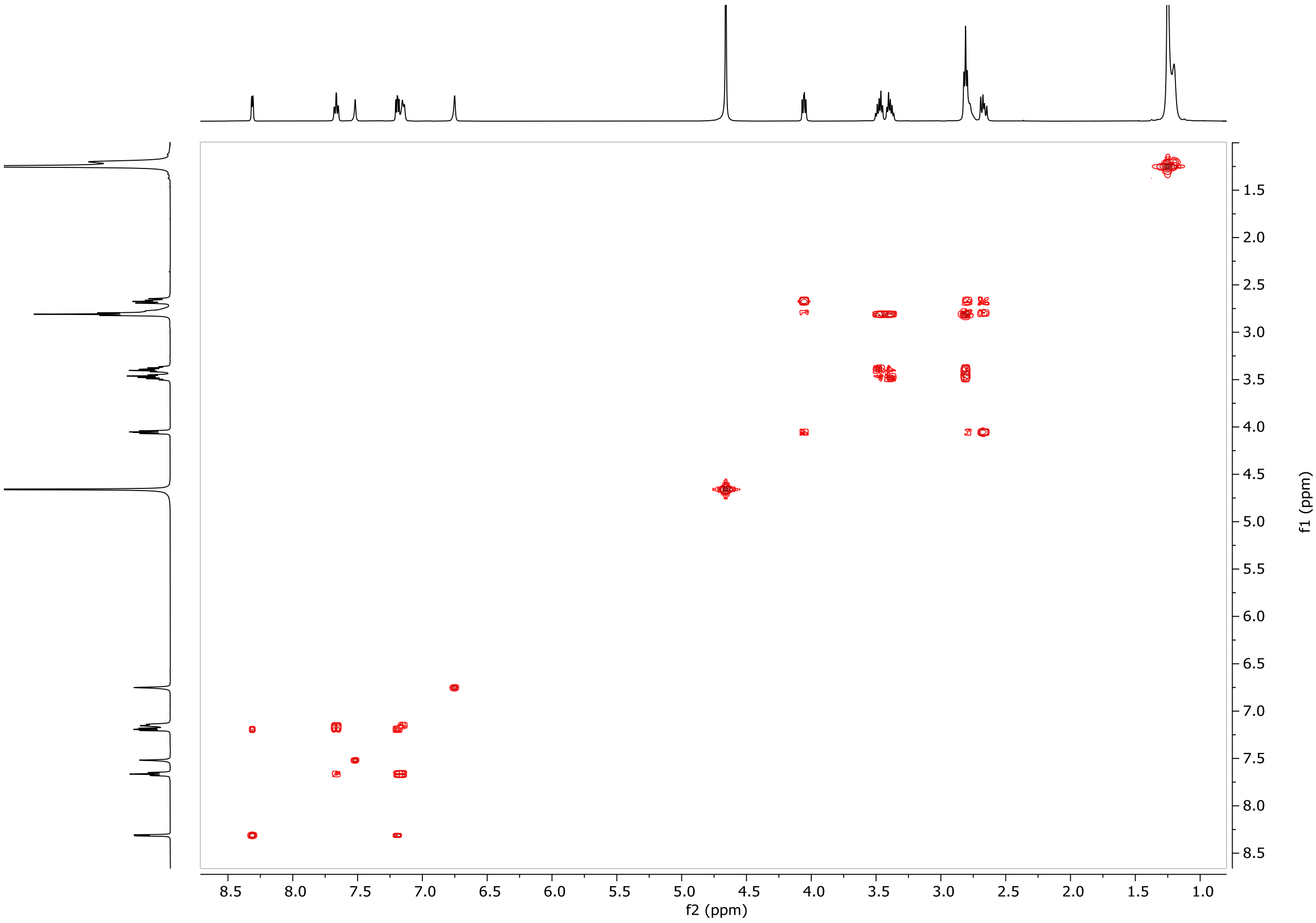

Figure S3. ^{1}H NMR COSY spectrum of L-BocHisPyr (D_2O, 500 MHz)

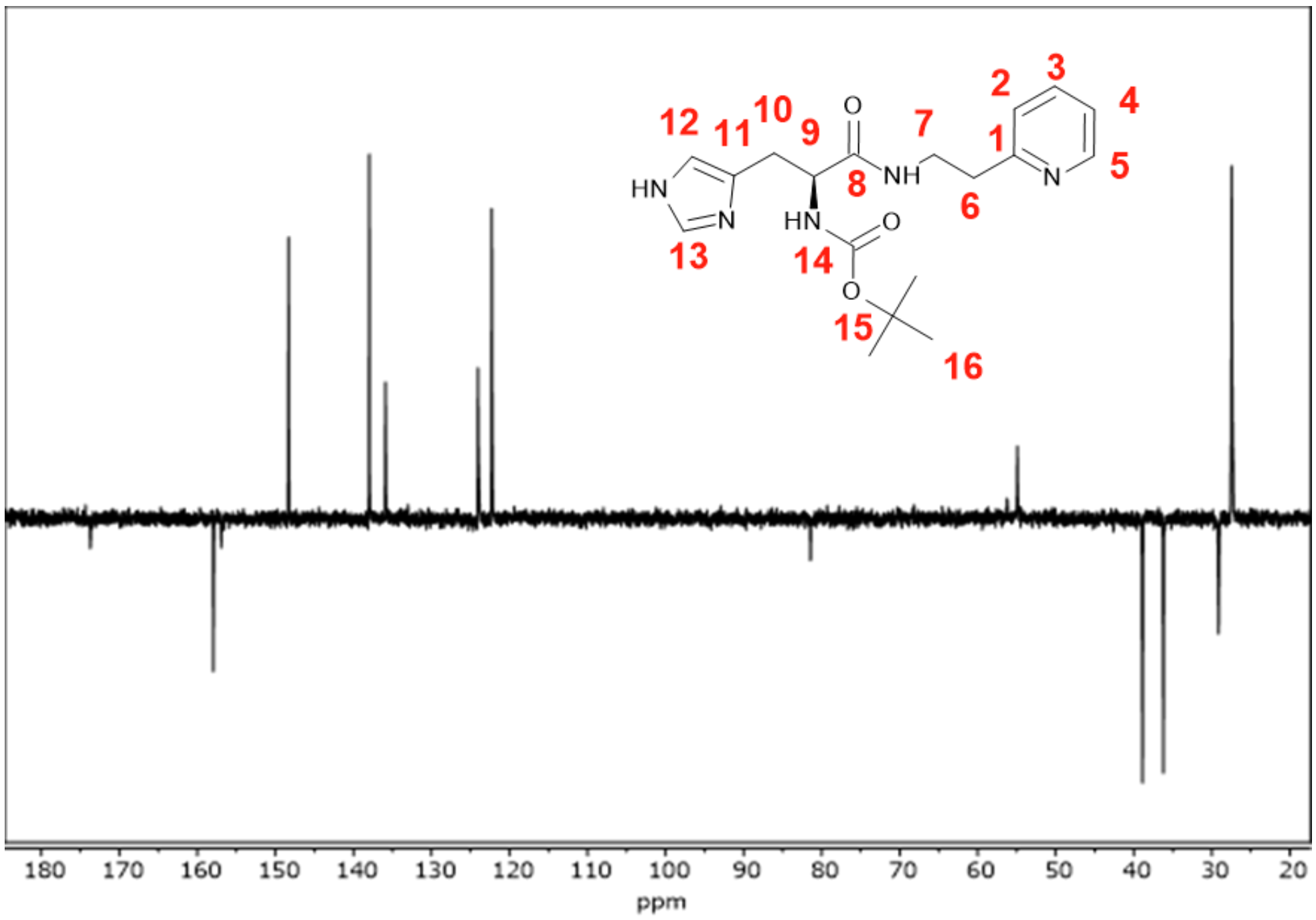

Figure S4. APT spectrum of L-BocHisPyr (D_2O, 125 MHz)

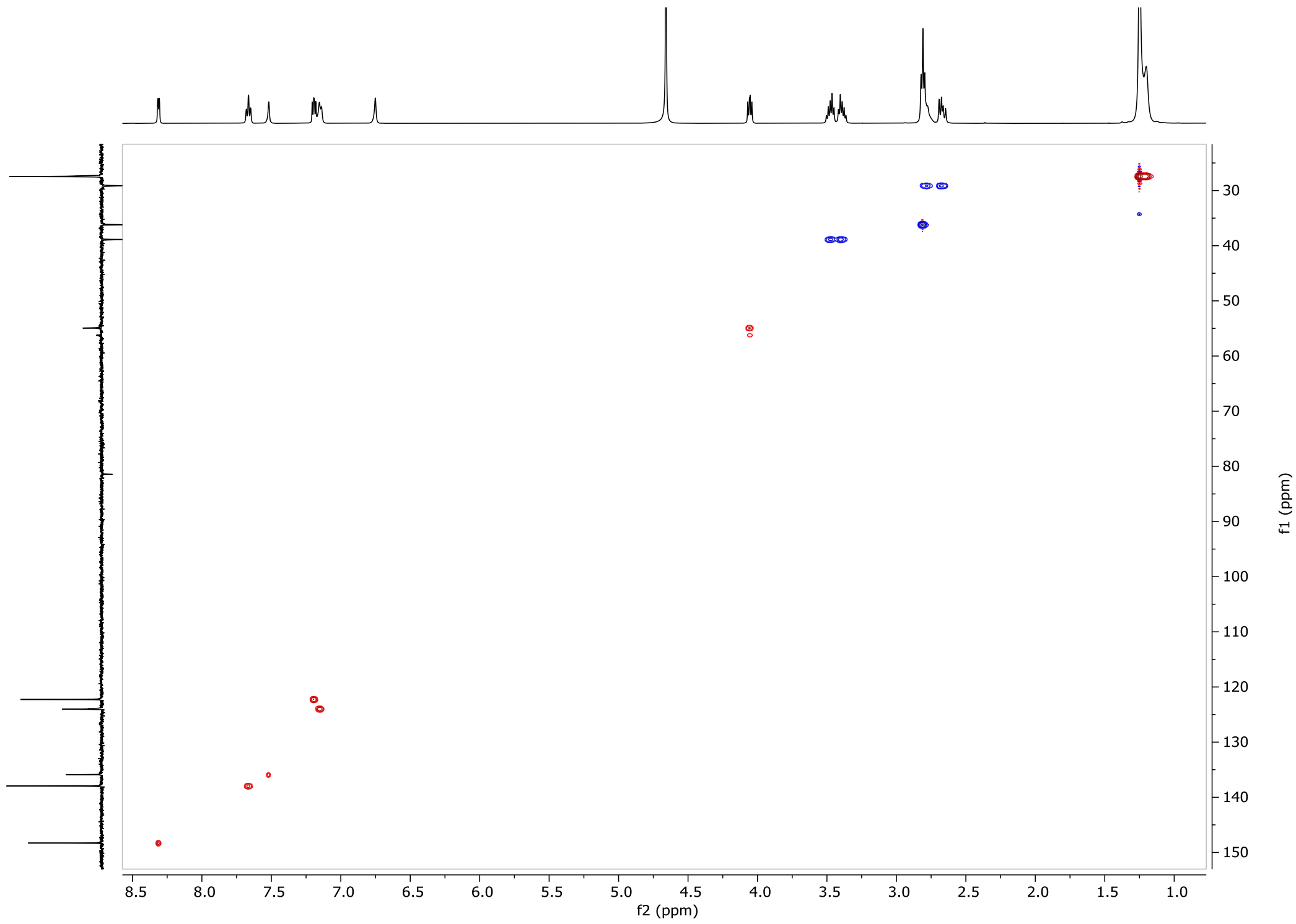

Figure S5. HSQC spectra of L-BocHysPyr (D_2O, 500 MHz)

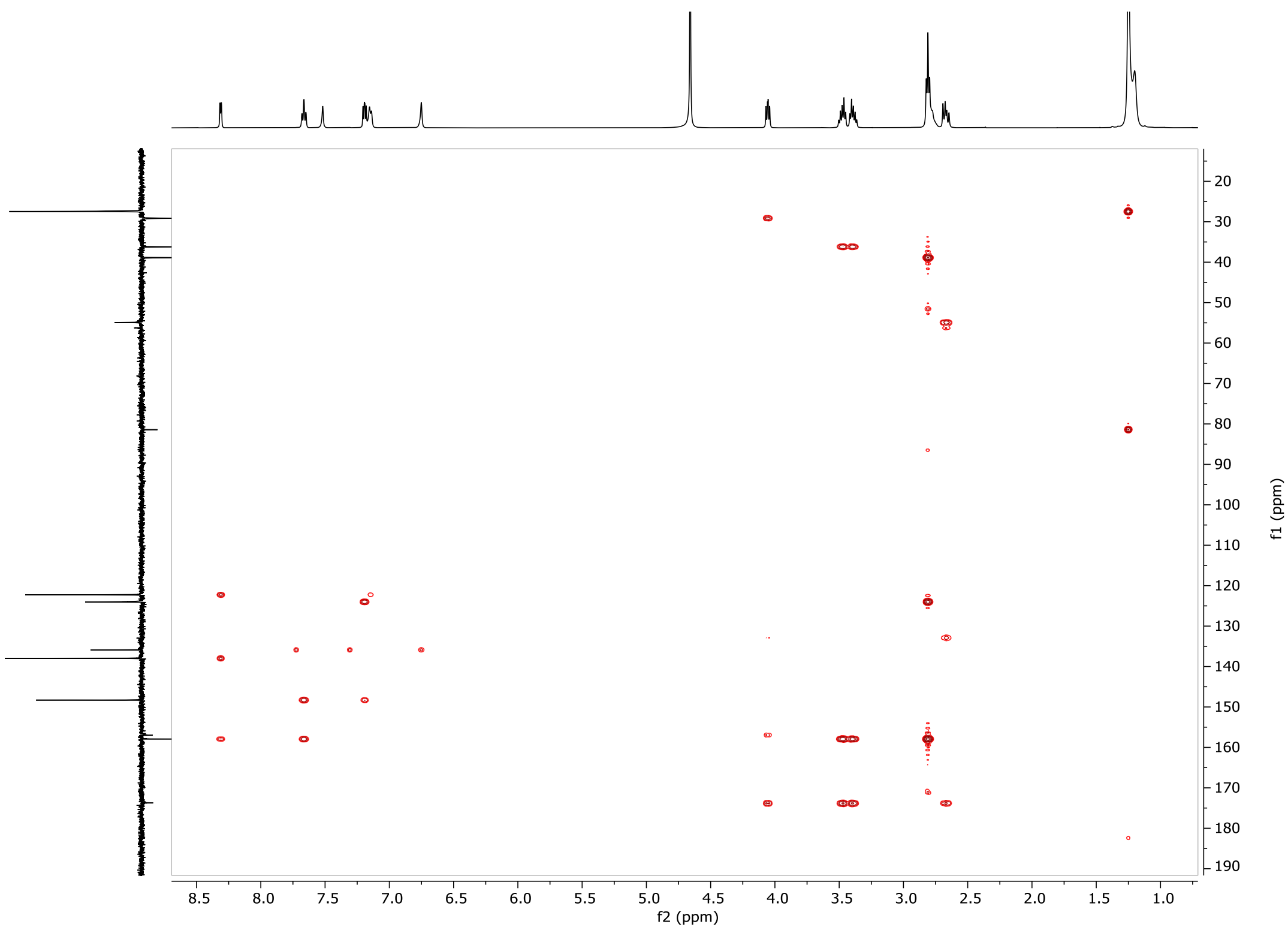

Figure S6. HMQC spectra of L-BocHisPyr (D_2O, 500 MHz)

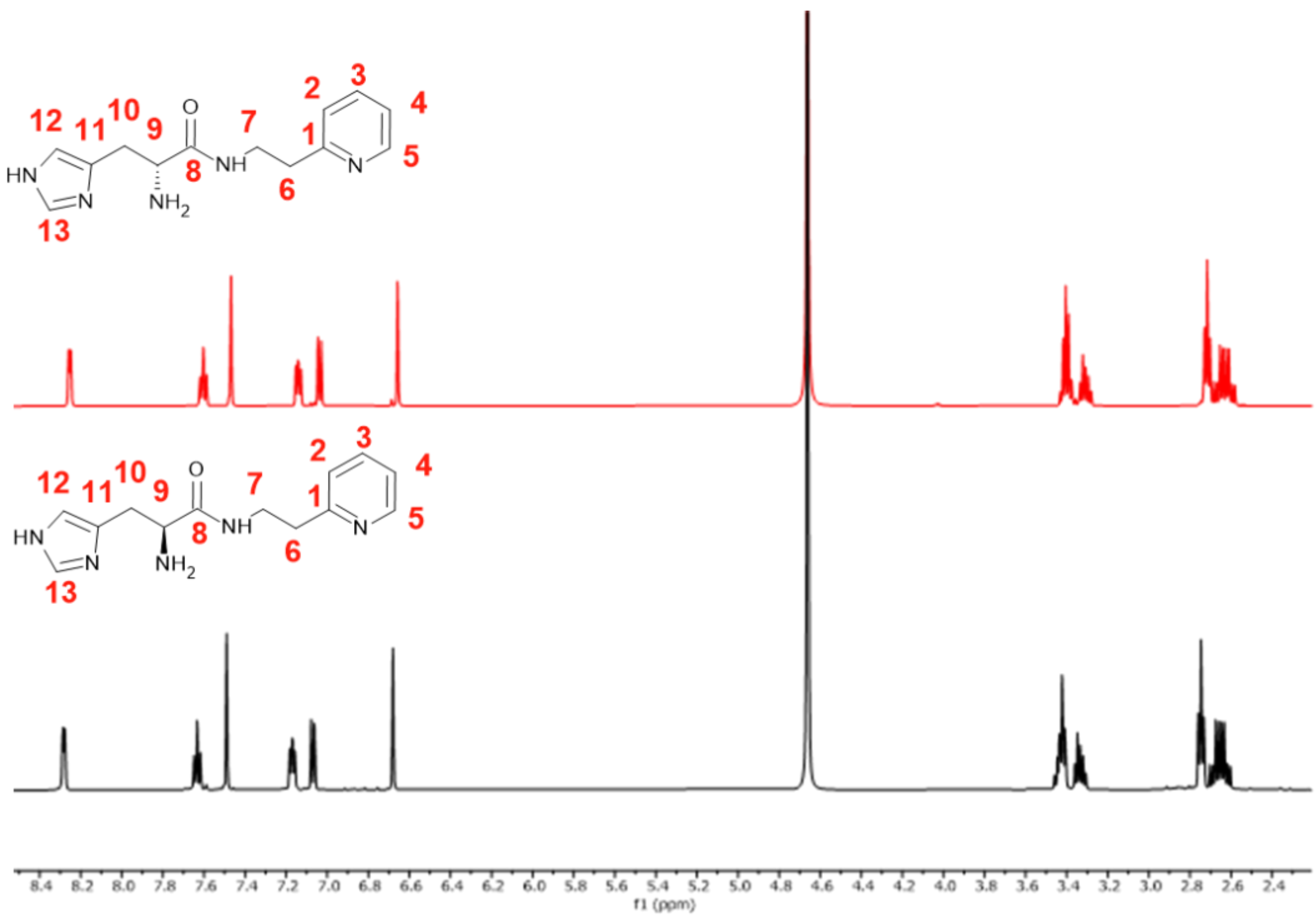

Figure S7. 1H NMR spectrum of D-HisPyr (red) and L-HisPyr (black) (D_2O, 500 MHz)

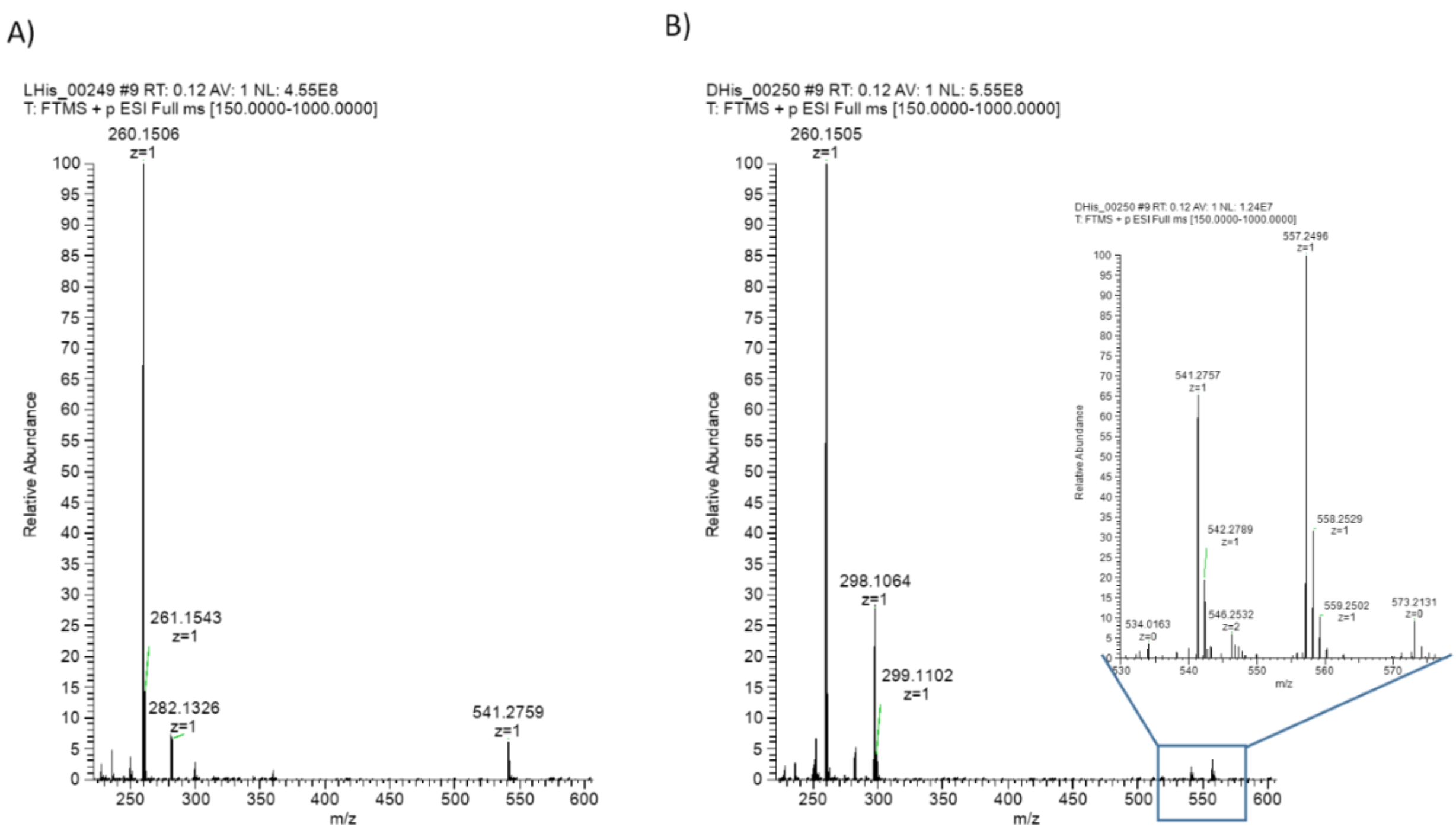

Figure S8. HR ESI spectra of L-HisPyr (A) and D-HisPyr (B).

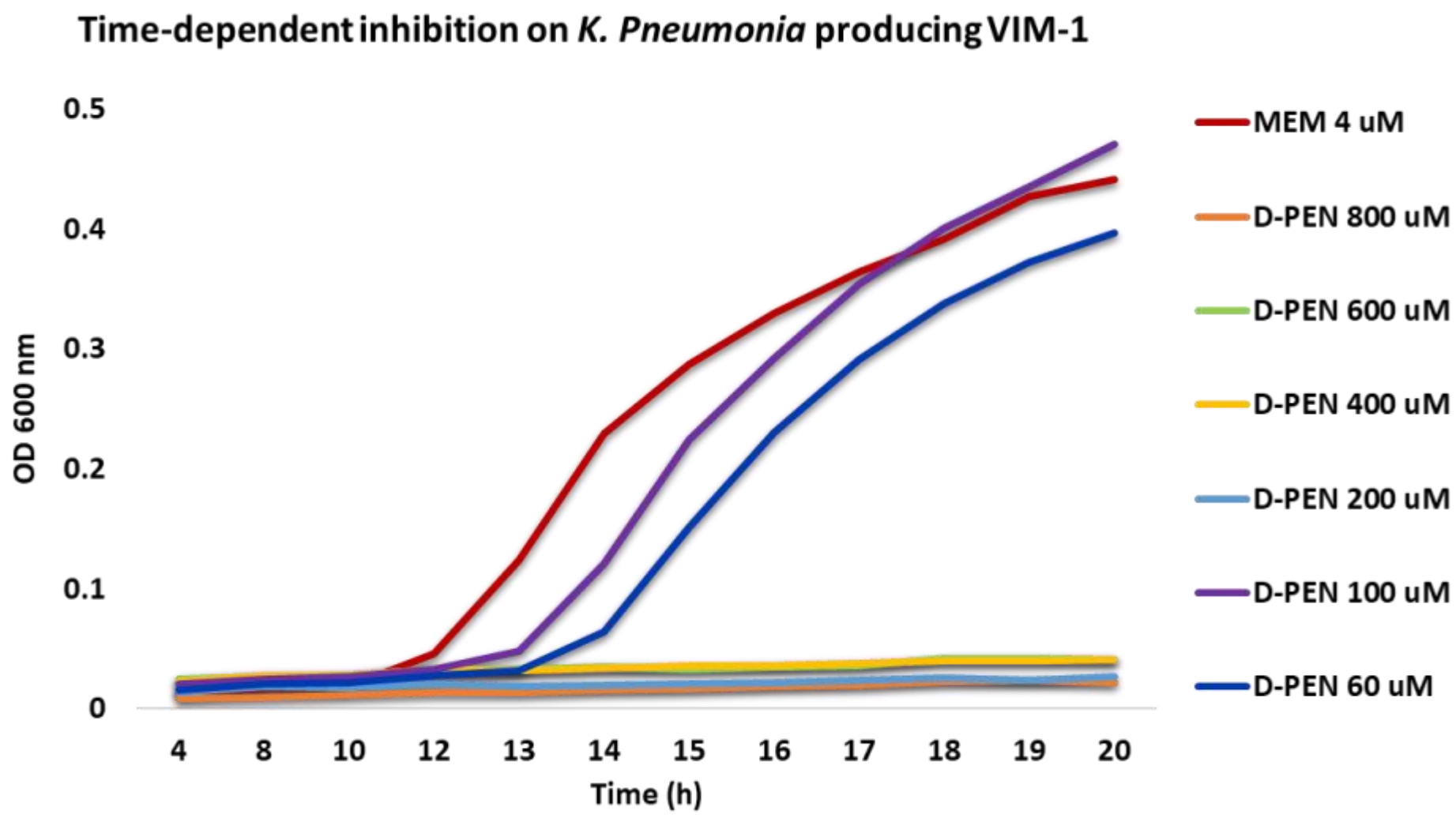

Figure S9. Time-dependent inhibition of K. Pneumonia-producing VIM-1 using different concentrations of D-Pen co-administered with MEM (4 μM)

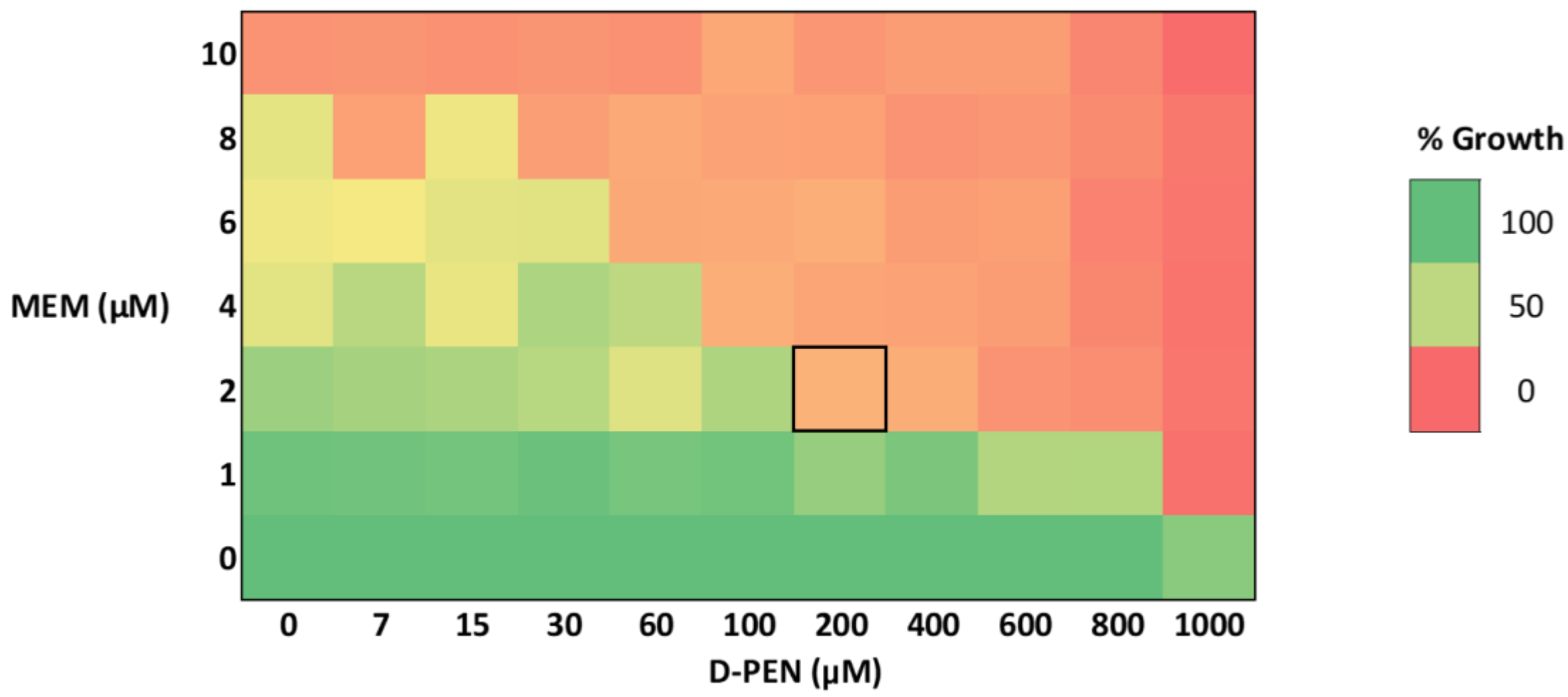

Figure S10. Checkerboard assay of MEM with D-PEN

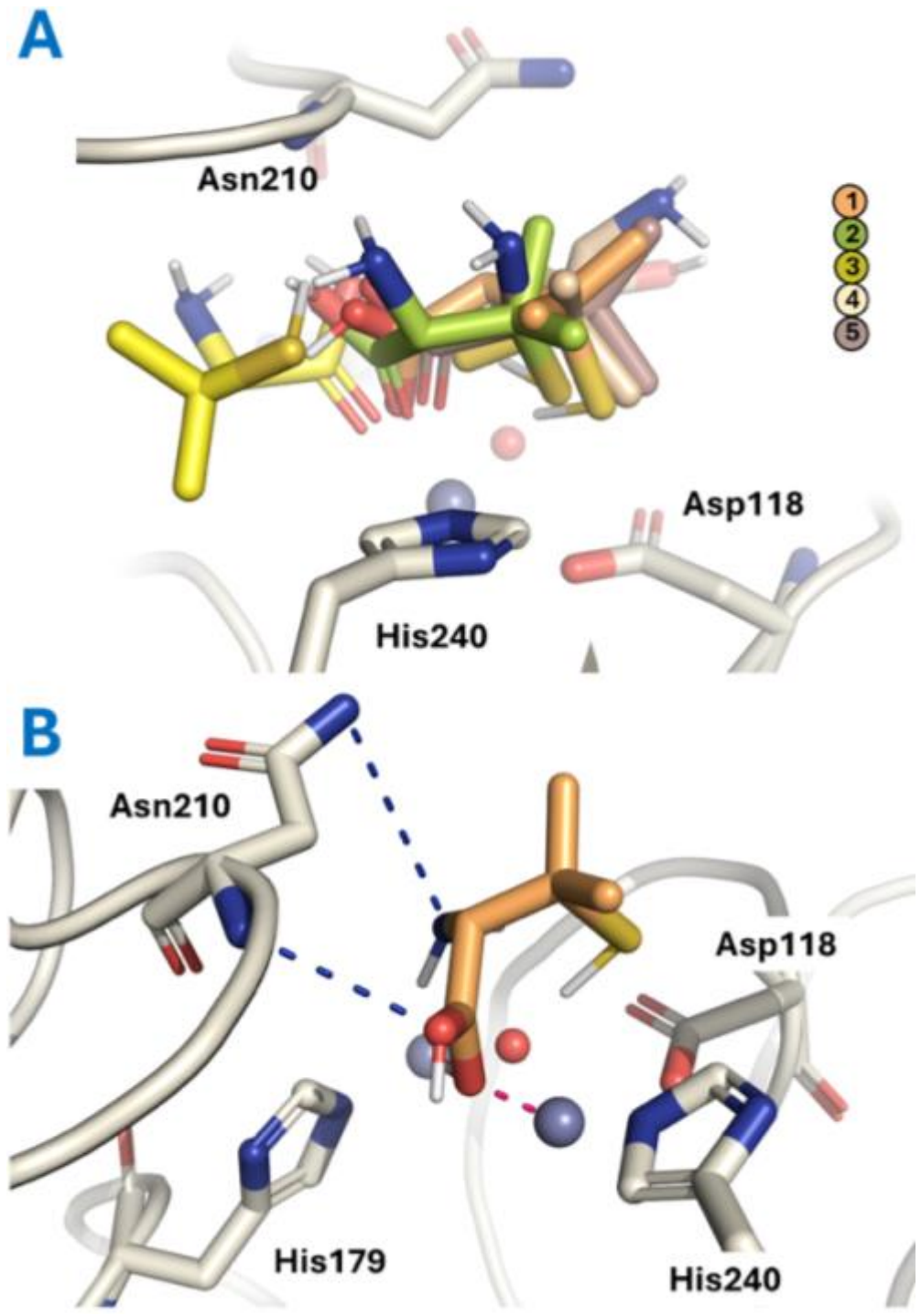

Figure S11. Docking poses of D-pen in the active site of VIM-1. (A) Overlay of the top five docking poses, showing a highly similar binding orientation across all poses. (B) Close-up view of the top-ranked pose, highlighting coordination of the catalytic Zn^{2+} ion (grey sphere) primarily through the thiol and carboxylate groups of D-Pen.

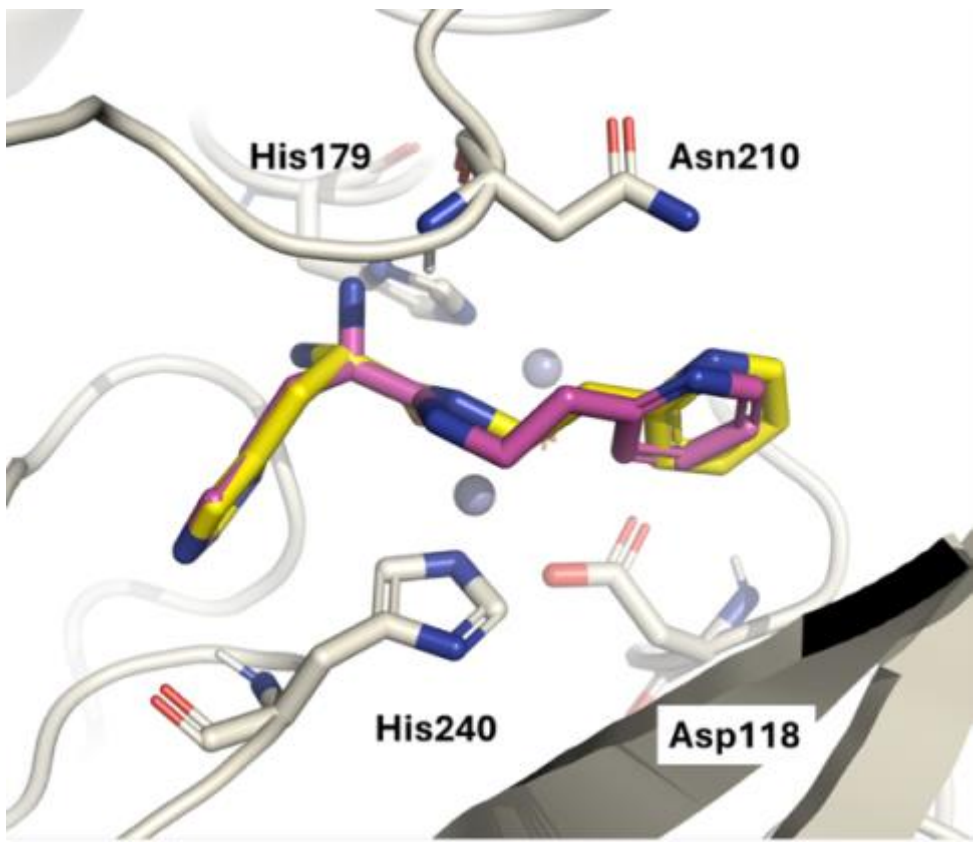

Figure S12. Overlay of the docked binding poses of the two enantiomers of HisPyr within the active site of VIM-1. The L-enantiomer is shown in yellow, while the D-enantiomer is shown in magenta. Both ligands adopt comparable orientations within the catalytic pocket and interact with the Zn^{2+} center and surrounding residues, highlighting their similar binding modes despite opposite chirality. Key active-site residues and metal ions are shown in stick representation, with Zn^{2+} depicted as a sphere.

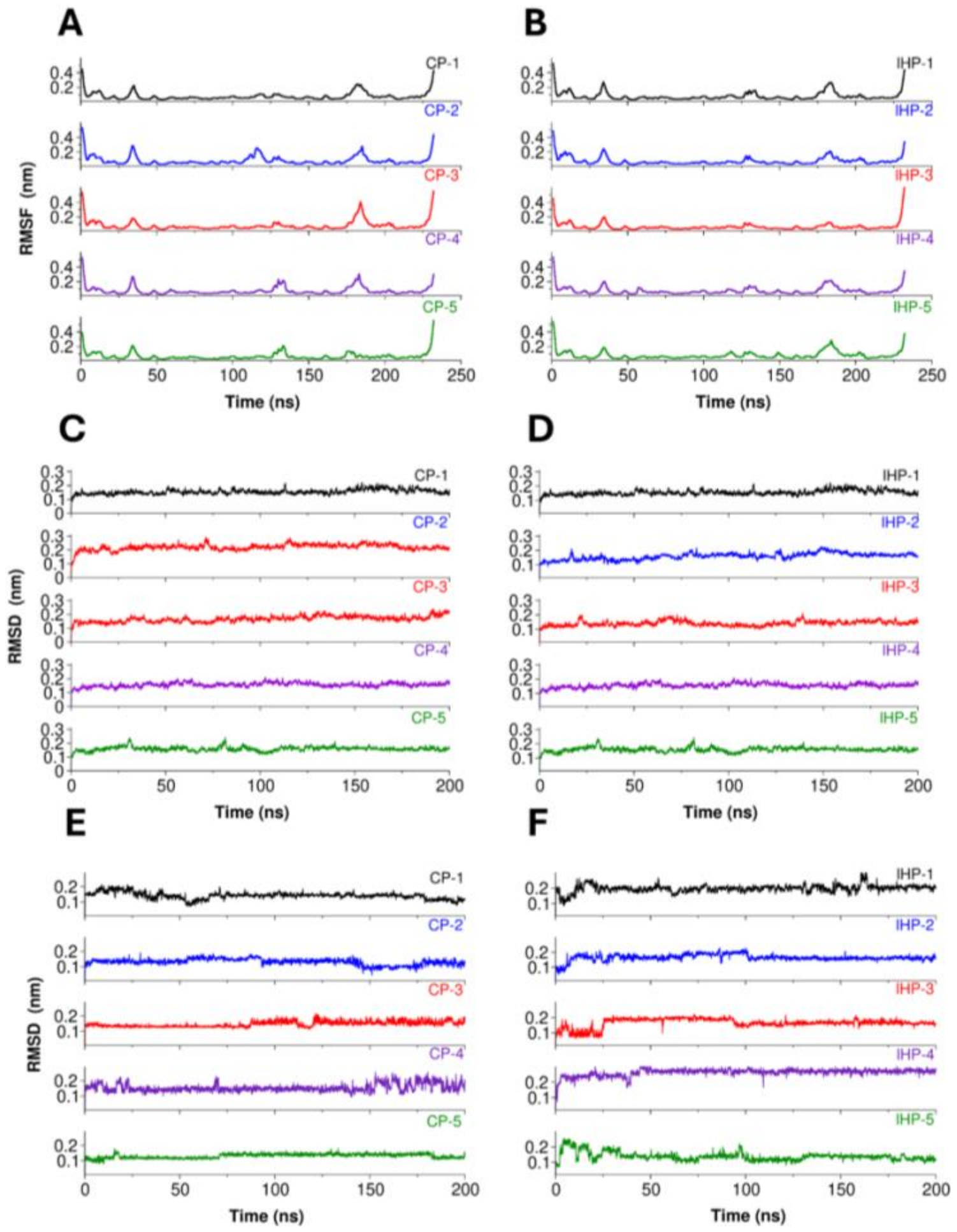

Figure S13. Structural stability analysis of the MD simulations of the VIM-1–CP and VIM-1–L-HisPyr complexes across five independent simulations (CP-1 to CP-5 and L-HisPyr-1 to L-HisPyr-5). (A-B) Root mean square fluctuation (RMSF) of protein residues; (C-D) RMSD of the protein Cα atoms (E-F) and of the ligand heavy atoms as a function of time.

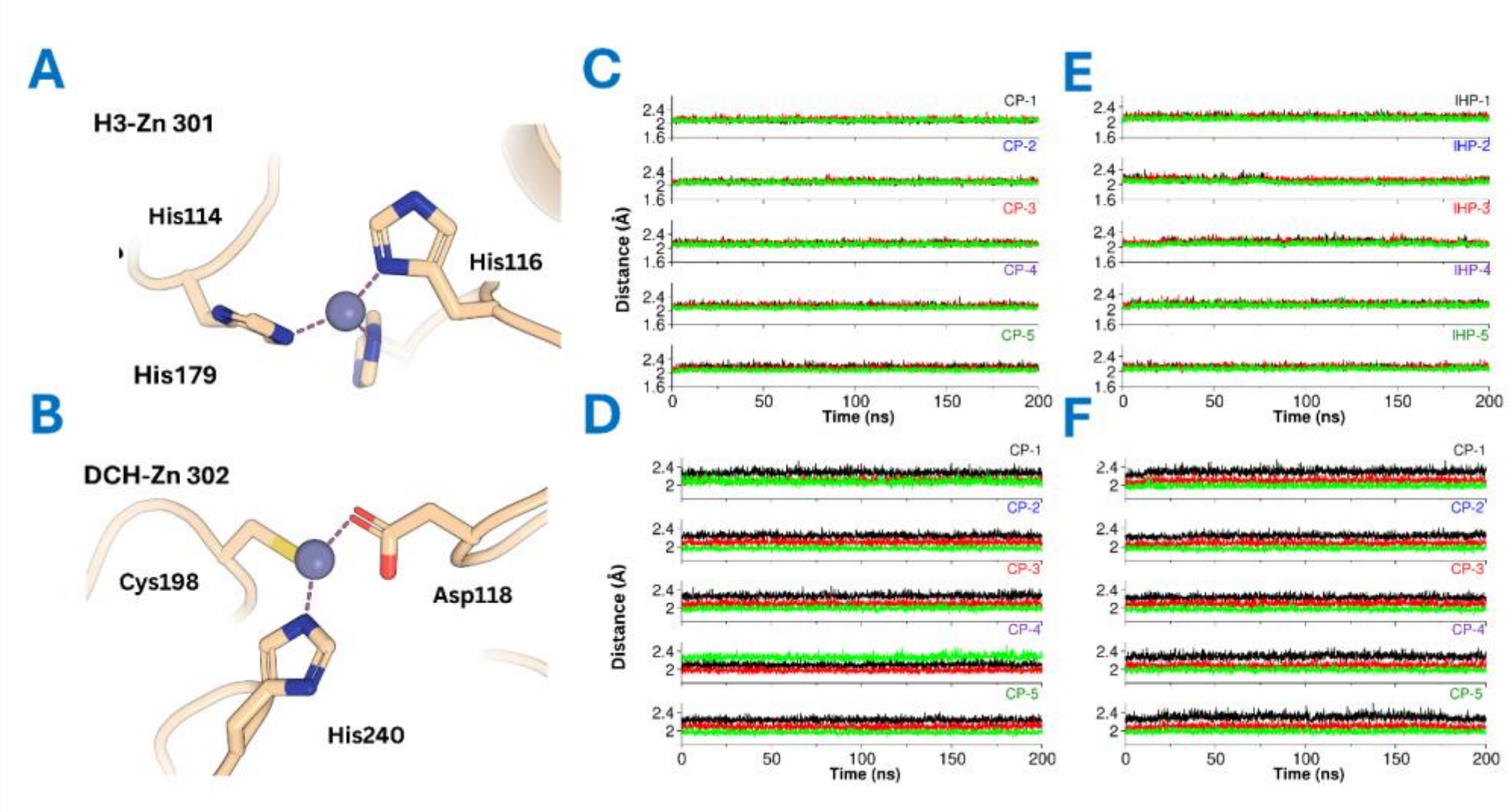

Figure S14. Close up views of the Zn^{2+} ions (Zn301 at the H3 site and Zn302 at the DCH site) along with key coordinating residues (A, B). The distances (nm) between Zn^{2+} ions and the coordinating residues from the H3 (C, E) and DCH (D, F) clusters across five independent MD simulations.

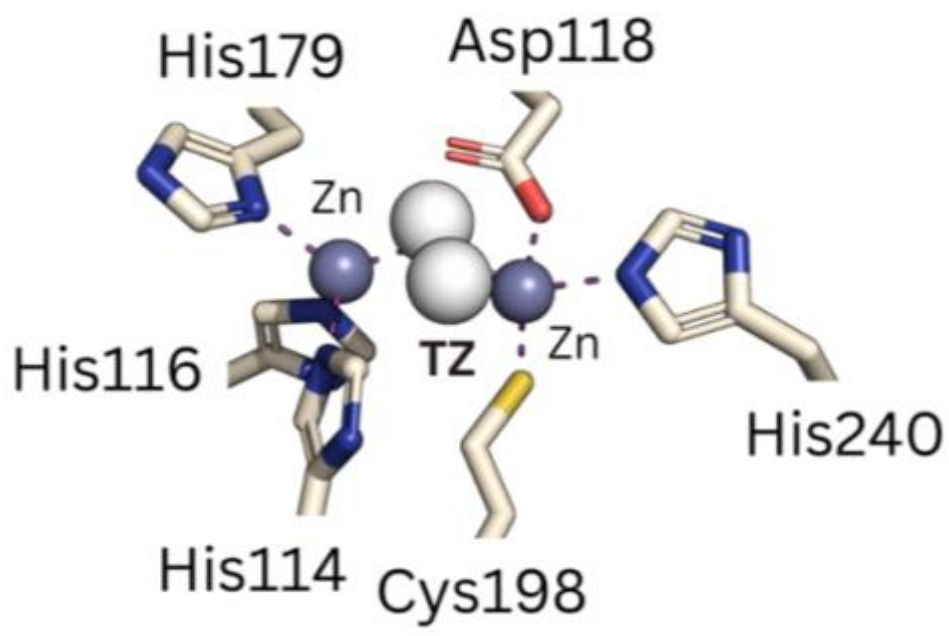

Figure S15. Active site of VIM-1 highlighting key residues involved in zinc coordination. The Zn^{2+} ions are coordinated by His114, His116, His179, His240, Asp118, and Cys198. TZ pseudoatoms (white spheres) were introduced using the *zinc_pseudo.py* script to improve the representation of metal coordination and enhance docking accuracy in AutoDock4Zn.

Table S1. Species found and calculated for the L- and D-HisPyr conjugates in ESI-HRMS.

**Compound(L)**	**Species**	**Formula**	**Theoretical (m/z)**	**Observed (m/z)**	**Error (ppm)**
**L-HisPyr**	$[L+H]^+$	$C_{13}H_{18}N_5O$	260.1506	260.1506	0.00
	$[L+Na]^+$	$C_{13}H_{17}N_5ONa$	282.1325	282.1326	0.35
	$[2L+Na]^+$	$C_{26}H_{34}N_{10}O_2Na$	541.2758	541.2759	0.18
**D-HisPyr**	$[L+H]^+$	$C_{13}H_{18}N_5O$	260.1506	260.1505	-0.38
	$[L+K]^+$	$C_{13}H_{17}N_5OK$	298.1325	298.1064	-0.34
	$[2L+Na]^+$	$C_{26}H_{34}N_{10}O_2Na$	541.2758	541.2757	-0.18
	[2L+K]+	$C_{26}H_{34}N_{10}O_2K$	557.2497	557.2496	-0.18

Table S2: Docking scores and ligand efficiency of selected ligands. The docking scores represent the binding affinity between each ligand and VIM-1, with lower values indicating stronger binding. The top five ranked poses for each ligand are reported.

Ligand	Pose Rank	Docking Score (kcal/mol)
CP	1	-7.1
	2	-7.0
	3	-6.9
	4	-6.9
	5	-6.8
D-Pen	1	-4.0
	2	-3.8
	3	-3.7
	4	-3.6
	5	-3.6
D-HisPyr	1	-7.3
	2	-6.8
	3	-6.3
	4	-6.1
	5	-6.0
L-HisPyr	1	-7.2
	2	-6.9
	3	-5.9
	4	-5.8
	5	-5.7

Table S3. PLIP-identified interactions between Chlorporpamide (CP) and VIM-1 across docking poses CP-1–CP-5.

Interaction type	VIM-1 residue	$d_{D–A}$ (Å)	$d_{H–A}$ (Å)	$\widehat{D\text{-}H\text{-}A}$ (°)	Offset[1] (Å)	Geometry or donor assignment
**Pose: CP-1**						
Hydrophobic	Trp87	3.26	—	—	—	Nonpolar contact
Hydrophobic	His116	3.41	—	—	—	Nonpolar contact
Hydrophobic	Asp117	3.99	—	—	—	Nonpolar contact
H-bond	Asp118	3.85	3.05	139.02	—	Ligand donor; protein acceptor
H-bond	Asn210	3.16	2.33	141.63	—	Protein donor; ligand acceptor
H-bond	Asn210	2.85	1.93	155.12	—	Protein donor; ligand acceptor
π–π stacking	Phe62	5.00	—	25.67	0.64	Parallel
**Pose: CP-3**						
Hydrophobic	His116	3.35	—	—	—	Nonpolar contact
Hydrophobic	Asp117	3.80	—	—	—	Nonpolar contact
H-bond	Asp118	2.98	2.10	147.90	—	Ligand donor; protein acceptor
H-bond	Asp118	3.90	3.18	130.50	—	Ligand donor; protein acceptor
H-bond	Asn210	2.83	1.87	166.47	—	Protein donor; ligand acceptor
π–cation	His240	3.77	—	—	0.39	Protein cation–ligand ar. ring
**Pose: CP-4**						
Hydrophobic	Trp87	3.76	—	—	—	Nonpolar contact
Hydrophobic	His116	3.87	—	—	—	Nonpolar contact
Hydrophobic	Glu146	3.86	—	—	—	Nonpolar contact
Hydrophobic	Asn210	3.77	—	—	—	Nonpolar contact
H-bond	His116	3.82	3.18	124.79	—	Ligand donor; protein acceptor
H-bond	Asp117	2.84	1.90	158.03	—	Protein donor; ligand acceptor
H-bond	Asp118	2.88	2.08	137.15	—	Protein donor; ligand acceptor
H-bond	Asp118	2.56	2.14	105.79	—	Protein donor; ligand acceptor
**Pose: CP-5**						
Hydrophobic	His116	3.64	—	—	—	Nonpolar contact
H-bond	Asp117	3.10	2.70	104.81	—	Protein donor; ligand acceptor
H-bond	Asp118	2.83	2.05	134.84	—	Ligand donor; protein acceptor
H-bond	Asp118	3.91	3.22	128.16	—	Ligand donor; protein acceptor
H-bond	Asp118	2.97	2.08	148.42	—	Protein donor; ligand acceptor
H-bond	Asp118	2.64	2.13	113.28	—	Protein donor; ligand acceptor
H-bond	Asn210	3.11	2.41	127.90	—	Protein donor; ligand acceptor
π–π stacking	His116	3.56	—	12.63	0.41	Parallel

Notes. The interaction-distance column reports the atom-atom distance for hydrophobic contacts, donor-acceptor (D-A) distance for H-bonds, ring-centroid distance for π-π stacking, and charge-centre-ring-centroid distance for π-cation interactions. For H-bonds, the angle is the donor angle; for π-π stacking, it is the interplanar angle. lHP-1 and lHP-2 produced similar PLIP interaction profiles. [1] Offset between the interacting groups. See PLIP documentation for more information. See Figure 6 in the main manuscript for visual representation of these interactions.

Abbreviations: D-A, donor-acceptor; H-A, hydrogen-acceptor; —, not applicable.

Table S4. PLIP-identified interactions between L-HisPyr and VIM-1 across docking poses lHP-1–lHP-5.

Interaction type	VIM-1 residue	$d_{D–A}$ (Å)	$d_{H–A}$ (Å)	$\widehat{D\text{-}H\text{-}A}$ (°)	Offset[1] (Å)	Geometry or donor assignment
**Pose: lHP-1**						
Hydrophobic	Trp87	3.53	—	—	—	Nonpolar contact
Hydrophobic	Asp118	3.91	—	—	—	Nonpolar contact
Hydrophobic	Asn210	3.16	—	—	—	Nonpolar contact
H-bond	Cys198	3.43	2.66	136.08	—	Ligand donor; protein acceptor
H-bond	His201	3.18	2.21	159.79	—	Ligand donor; protein acceptor
H-bond	Asn210	2.81	2.07	131.02	—	Protein donor; ligand acceptor
H-bond	Asn210	3.60	2.65	161.71	—	Protein donor; ligand acceptor
H-bond	Gly239	3.97	3.62	103.93	—	Protein donor; ligand acceptor
H-bond	His240	3.14	2.38	133.15	—	Protein donor; ligand acceptor
π–π stacking	Phe62	4.55	—	62.83	0.84	T-shaped
π–π stacking	His201	4.22	—	63.50	1.37	T-shaped
π–π stacking	His240	4.46	—	74.03	0.89	T-shaped
π–cation	His116	5.59	—	—	1.17	Protein cation–ligand ar. ring
**Pose: lHP-3**						
Hydrophobic	Tyr67	3.34	—	—	—	Nonpolar contact
Hydrophobic	His240	3.70	—	—	—	Nonpolar contact
H-bond	Cys198	3.44	2.65	137.32	—	Ligand donor; protein acceptor
H-bond	His201	3.17	2.19	158.42	—	Ligand donor; protein acceptor
H-bond	Asn210	3.33	2.39	158.48	—	Protein donor; ligand acceptor
H-bond	Gly239	3.98	3.63	103.73	—	Protein donor; ligand acceptor
H-bond	His240	3.14	2.38	133.27	—	Protein donor; ligand acceptor
π–π stacking	His201	4.21	—	62.59	1.48	T-shaped
π–π stacking	His240	4.49	—	75.27	0.84	T-shaped
**Pose: lHP-4**						
Hydrophobic	Trp87	3.44	—	—	—	Nonpolar contact
H-bond	Asp117	3.40	2.58	141.09	—	Protein donor; ligand acceptor
H-bond	Asn210	2.87	2.02	143.80	—	Protein donor; ligand acceptor
H-bond	Asn210	2.97	1.99	176.12	—	Protein donor; ligand acceptor
π–cation	His240	4.40	—	—	1.36	Protein cation–ligand ar. ring
**Pose: lHP-5**						
Hydrophobic	Pro68	3.71	—	—	—	Nonpolar contact
Hydrophobic	His240	3.33	—	—	—	Nonpolar contact
H-bond	His179	2.82	2.02	133.52	—	Ligand donor; protein acceptor
H-bond	Asn210	2.87	1.98	148.98	—	Protein donor; ligand acceptor
H-bond	Gly239	3.28	2.79	111.39	—	Protein donor; ligand acceptor
H-bond	His240	3.08	2.24	142.68	—	Protein donor; ligand acceptor
π–π stacking	His201	4.19	—	72.27	1.22	T-shaped
π–π stacking	His240	4.51	—	66.60	1.31	T-shaped
π–π stacking	His240	4.70	—	24.81	1.98	Parallel

Notes. The interaction-distance column reports the atom-atom distance for hydrophobic contacts, donor-acceptor (D-A) distance for H-bonds, ring-centroid distance for π-π stacking, and charge-centre-ring-centroid distance for π-cation interactions. For H-bonds, the angle is the donor angle; for π-π stacking, it is the interplanar angle. lHP-1 and lHP-2 produced similar PLIP interaction profiles.[1] Offset between the interacting groups. See PLIP documentation for more information. See Figure 6 in the main manuscript for visual representation of these interactions.

Abbreviations: D-A, donor-acceptor; H-A, hydrogen-acceptor; —, not applicable.
